# Multi-omics integration reveals pan-cancer roles of *ZIC* family genes in prognosis, immune microenvironment remodeling, and therapeutic vulnerability

**DOI:** 10.1007/s12672-026-05100-2

**Published:** 2026-04-28

**Authors:** Pengyi Ma, Feijuan Zhang, Pengchai Ma

**Affiliations:** 1 Tianjin Key Laboratory of Cellular and Molecular Immunology and Key Laboratory of the Educational Ministry of China, Tianjin Medical University, Tianjin, China; 2 Department of Respiratory, The Children’s Hospital of Tianjin (Children’s Hospital of Tianjin University), Tianjin, China; 3 Department of Rheumatology and Clinical Immunology, Tianjin Children’s Hospital, Tianjin, 300134 China

**Keywords:** Pan-cancer analysis, *ZIC* genes, Single-cell sequencing, Disease biomarkers

## Abstract

**Background:**

The *ZIC* gene family (*ZIC1*–*ZIC5*) encodes zinc-finger transcription factors with established roles in neural development. However, a systematic pan-cancer analysis integrating their multi-omics landscapes, clinical prognostic value, and immunomodulatory mechanisms remains unexplored, limiting their potential as cross-cancer biomarkers.

**Methods:**

We performed a comprehensive multi-omics analysis across 33 cancer types, assessing *ZIC1*-*ZIC5* gene expression, prognostic significance, genomic alterations (somatic mutations, copy number variations), and immune interactions (immune infiltration, checkpoint genes, microsatellite instability, and tumor mutation burden). Drug sensitivity analysis (validated by CCK-8 assays) and immunotherapy cohort validation were conducted, with focused immune infiltration assessment in liver hepatocellular carcinoma (LIHC). Additionally, using qRT-PCR and Western blot, we assessed the expression of *ZIC2* in tissues from hepatocellular carcinoma patients and investigated the transcriptional networks of *ZIC* genes across various cell populations within the tumor microenvironment.

**Results:**

*ZIC* genes exhibited cancer-type-specific dysregulation, significantly correlating with patient survival, genomic instability, and immune cell infiltration. Drug response analysis identified *ZIC* expression as a potential predictive biomarker, further corroborated by CCK-8 results confirming the therapeutic vulnerability of *ZIC2*-high HCC cells to alisertib. Notably, distinct *ZIC* signatures were linked to immunotherapy outcomes, as supported by GSEA-enriched immune pathways. In LIHC, *ZIC* alterations were closely associated with immune microenvironment dynamics, corroborated by protein-level validation.

**Conclusion:**

This study identified *ZIC* family genes as multi-functional regulators in pan-cancer contexts, serving as promising prognostic biomarkers and therapeutic targets across cancers, particularly in LIHC. Their immune-modulatory functions highlight their potential utility in immunotherapy optimization, providing a foundation for future translational research in precision oncology.

**Supplementary Information:**

The online version contains supplementary material available at 10.1007/s12672-026-05100-2.

## Introduction

Cancer is a substantial worldwide public health concern, marked by its gradual progression and elevated mortality rates, despite notable advancements in detection and treatment. In China, it ranks among the leading causes of death, with an estimated 4.064 million new cases and 2.4135 million deaths recorded in 2016 [1]. Among various cancer types, lung, colorectal, gastric, liver, and breast cancer stand out as the five primary contributors to the disease burden, collectively responsible for 57.4% of new diagnoses. In contrast, lung, liver, gastric, colorectal, and esophageal cancers account for 69.3% of cancer-related deaths. As a genomic disease, cancer development and progression are potentially linked to complex genetic alterations, necessitating deeper molecular insights to improve prevention and therapeutic strategies.

Cancers across different organs often exhibit shared traits, and common molecular patterns allow insights from one cancer type to benefit others [2]. Pan-cancer analysis, which investigates multiple cancer types, has illuminated critical dysregulated biological processes across diverse cancer lineages. This approach deepens our understanding of cancer biology and medicine while unveiling new therapeutic possibilities [3].The Cancer Genome Atlas (TCGA) project, having molecularly profiled over 20,000 primary samples from 33 cancer types, has markedly advanced this field, offering a systematic view of cancer mechanisms at genomic, epigenomic, transcriptomic, proteomic, and clinical levels [4]. By integrating multi-omics data, researchers can uncover dynamic, tissue-specific gene expression patterns, facilitating the advancement of precision medicine.

Recent research has revealed the striking functional dualism of *ZIC* family members in carcinogenesis, characterized by their ability to transition between oncogenic and tumor-suppressive roles depending on the histological context. For instance, while *ZIC2* is a well-established oncogene in liver and colorectal cancers [5, 6], it has been reported to be downregulated and serve a potential protective role in breast cancer [7]. Such functional inconsistencies highlight the complex regulatory mechanisms dictated by specific cancer types and microenvironmental conditions, positioning the *ZIC* family as high-value yet enigmatic therapeutic targets.

The *ZIC* family, comprising five members (*ZIC1*–*ZIC5*), plays multifaceted roles across vertebrate development and carcinogenesis. While these members share conserved zinc-finger domains, their functional impacts are highly context-dependent, often exhibiting a dichotomy between oncogenic and tumor-suppressive characteristics. The specific biological functions and cancer-related roles of individual *ZIC* family members, along with their associated mechanisms, are summarized in Table 1 [8–16]. Collectively, these studies suggest that the *ZIC* family represents a group of critical biomarkers and potential therapeutic targets.

**Table 1:** Functional roles of the *ZIC* family in development and cancer

Gene	Normal development roles	Roles in cancer	References
*ZIC1*	Cerebellar granule cell proliferation, neural plate and spinal cord patterning	Oncogenic in LGG; Tumor-suppressive in gastric cancer	[8–10]
*ZIC2*	Forebrain and neural crest development, neural tube patterning	Oncogenic in ovarian cancer and LIHC	[11, 12]
*ZIC3*	Early embryonic patterning, heart development, neural tube closure	Oncogenic (linked to better survival when lowly expressed in GBM)	[9, 13]
*ZIC4*	Forebrain and midbrain neural development	Diagnostic and prognostic biomarkers in LIHC	[14]
*ZIC5*	Neural crest development, cerebellar and neural structure formation	Oncogenic in NSCLC and CESC	[15, 16]

LGG, brain lower-grade glioma; GBM, glioblastoma multiforme; NSCLC, non-small cell lung cancer; CESC, cervical squamous cell carcinoma and endocervical adenocarcinoma

Although direct research connecting the *ZIC* family to the immune system is sparse, emerging evidence indicates that the *ZIC* family may have an immunomodulatory function in cancer. *ZIC1* is expressed in immune cells and may regulate T-cell activation or differentiation [17]. In lung adenocarcinoma (LUAD), *ZIC2* overexpression correlates with infiltration of CD4 memory resting T cells, M1 macrophages, and plasma cells, potentially influencing tumor progression [18]. Notably, ZIC4 serves as an autoantigen in paraneoplastic neurological syndrome, with anti-ZIC4 antibodies triggering limbic encephalitis in small-cell lung cancer (SCLC) patients receiving atezolizumab [19]. Moreover, *ZIC5* expression positively correlates with immune cell infiltration levels in gliomas, suggesting its potential role in modulating the tumor immune microenvironment [9]. This immunomodulatory capacity might indirectly contribute to the tumor immune response by altering immune cell activity or the inflammatory conditions within the tumor microenvironment.

However, current research on the *ZIC* family remains largely piecemeal and scattered, often focusing on isolated genes without a holistic multi-omics perspective. Despite existing research on individual *ZIC* family members, a systematic pan-cancer investigation integrating multi-omics data to comprehensively evaluate their expression patterns, genomic alterations, and clinical significance remains unexplored. This study employs a multidimensional approach to analyze *ZIC* genes across 33 cancer types. Furthermore, while the *ZIC* family showed widespread significance across the pan-cancer cohort, we performed a focused deep dive into LIHC to explore the specific molecular mechanisms and clinical relevance of the *ZIC*-mediated immune landscape, supported by both single-cell transcriptomics and clinical sample validation. Through this integrative analysis, we aim to elucidate the biological roles of *ZIC* genes and provide translational insights for cancer diagnosis and therapy.

## Materials and methods

### Comprehensive pan-cancer data acquisition

Utilizing the TCGA dataset, we accessed comprehensive data including gene expression profiles, clinical information, survival statistics, mutation features, immune phenotypes, and stemness indices for 33 cancer types from the UCSC Xena database (http://xena.ucsc.edu/). Furthermore, quantile normalization was applied to microarray expression data retrieved from three Gene Expression Omnibus (GEO) immunotherapy (IT) studies: GSE215011 (*n* = 10), GSE223201 (*n* = 15), and GSE279750 (*n* = 10).

### Investigating *ZIC* gene family expression patterns across multiple cancer types

We retrieved gene expression data for 33 cancer types from TCGA, encompassing both tumor samples and, where available, adjacent normal samples. To visualize the expression profiles, we generated box plots illustrating the levels of five *ZIC* genes across tumor samples from these 33 cancer types. We utilized the Wilcoxon rank-sum test to assess the differences in *ZIC* gene expression among normal and tumor tissues for cancer types with available normal samples. Furthermore, we examined pairwise correlations among the five *ZIC* genes and visualized the resulting correlation matrix using the “corrplot” package (version 0.95) in R software (version 4.4.2).

### Evaluation of *ZIC* genes correlations with survival and genomic alterations

To determine the prognostic significance of *ZIC* expression levels, we performed survival analysis including overall survival (OS), disease-free survival (DFS), disease-specific survival (DSS), and progression-free survival (PFS) across 33 cancer types using the TCGA dataset. Survival outcomes were visualized using Kaplan–Meier curves and compared via the log-rank test. Furthermore, the association between *ZIC* expression and clinical characteristics—including tumor stage and treatment response—was evaluated.

The genomic landscape of *ZIC* genes was explored through cBioPortal, focusing on the frequency and types of copy number variations (CNVs) and single-nucleotide variants (SNVs). We further examined the correlation between *ZIC* mRNA levels and these genomic alterations. To assess the impact of genomic instability on prognosis, Kaplan–Meier analysis was utilized to compare the survival outcomes between patients with and without *ZIC* genomic alterations.

### Linking *ZIC* expression to TME, stemness indices, and immune subtypes

We investigated the correlation between *ZIC* gene expression and immune subtypes (C1–C6) across pan-cancer and LIHC cohorts. The ESTIMATE algorithm was employed to calculate immune and stromal scores, and their associations with *ZIC* expression levels were assessed using Spearman’s rank correlation analysis [20, 21]. Additionally, we evaluated the relationship between *ZIC* expression and cancer stemness using two distinct indices: DNA methylation-based stemness index (DNAss) and mRNA-based stemness index (RNAss). Spearman correlation was consistently applied to quantify these associations across the 33 TCGA tumor types.

### The correlation between immunological checkpoint gene (ICG) and *ZIC* gene expression levels, tumor mutational burden (TMB), and microsatellite instability (MSI)

We employed Spearman’s rank correlation to assess the association between *ZIC* gene expression levels and both TMB and MSI across 33 tumor types from the TCGA dataset [20, 21]. Heatmaps were generated to visualize these correlations using the “pheatmap” package in R.

### TIMER

Utilizing the Tumor Immune Estimation Resource 2.0 (TIMER 2.0, http://timer.comp-genomics.org/), we analyzed the correlation between *ZIC* gene expression and the infiltration levels of various immune cell (IC) types across multiple cancers.

### Examining drug sensitivity (DS) in contexts related to pan-cancer

Leveraging the CellMiner database (https://discover.nci.nih.gov/cellminer/home.do), we gathered DS and gene expression data from a shared set of samples. These samples originate from the NCI-60 panel—a collection of 60 human cancer cell lines representing nine distinct cancer types (leukemia, colon, lung, CNS, melanoma, ovarian, renal, prostate, and breast). Although the NCI-60 panel does not encompass all 33 cancer types analyzed in the TCGA, it remains the widely recognized standard for characterizing pan-cancer pharmacological responses and mechanistically linking gene expression to drug sensitivity. Following that, the DS data were filtered according to FDA standard accreditation and clinical laboratory validation. To evaluate the association between DS and *ZIC* gene expression levels, we applied the Pearson correlation test in our analysis, following established protocols for pharmacological response characterization [21].

### Predictive value of *ZIC* expression in IT cohorts

We obtained gene expression profiles and clinical data from three independent LIHC cohorts—GSE215011, GSE223201, and GSE279750—to assess the predictive value of *ZIC* gene expression in IT. Patients were classified as responders if they exhibited a complete response (CR) or partial response (PR) to IT, and as non-responders if they showed stable disease (SD) or progressive disease (PD), according to the Response Evaluation Criteria in Solid Tumors (RECIST).

### Gene set enrichment analysis (GSEA)

We used GSEA in our LIHC investigation to analyze the biological roles and putative signaling pathways linked to the *ZIC* genes [20, 21]. In our investigation, we employed gene sets from the Kyoto Encyclopedia of Genes and Genomes (KEGG), which were accessed via the Molecular Signatures Database (MSigDB).

### Clinicopathological and molecular feature analysis in LIHC

In LIHC, the association between *ZIC* expression and clinicopathological traits—including immune subtypes, disease stage, TNM classification, tumor differentiation, age, and sex—was analyzed using the Kruskal–Wallis test. Additionally, Spearman correlation was used to assess the relationship between *ZIC* expression and molecular markers such as RNAss, DNAss, MSI, and TMB.

### Impact of genomic alterations on immune infiltration in LIHC

The association between *ZIC* expression and the degree of immune infiltration in LIHC was examined using the TIMER 2.0 database. This investigation focused on six key types of immune cells: B cells, CD4 + T cells (Ths), CD8 + T cells (CTLs), macrophages (MΦs), neutrophils (PMNs), and dendritic cells (DCs). Furthermore, we employed TIMER 2.0 to analyze the associations between CNVs, *ZIC* mutation status, and the levels of these infiltrating immune cells.

### Experimental validation: clinical samples and qRT-PCR

Gene Expression Profiling Interactive Analysis (GEPIA2) [22], examines RNA-seq data from cancerous and healthy tissues sourced from TCGA and GTEx. We utilized this platform to assess the levels of pivotal genes in both malignant and benign samples across diverse human tissues.

The E.Z.N.A.TM Hp Total RNA Kit (OMEGA) was used to extract total RNA from 52 pairs of liver cancer tumors and surrounding tissues. The mRNA expression of *ZIC* in 52 LIHC specimens, compared with that in corresponding normal tissues, was determined via qRT‒PCR with the following primers: forward, 5′-GCGCAACTCCACAACCAGTA-3′; reverse, 5′- TGCCGCATATAGCGGAAAAAG-3′. *GAPDH* was used as the housekeeping gene, with the following primers: forward, 5′-GTCTCCTCTGACTTCAACAGCG-3′; reverse, 5′- ACCACCCTGTTGCTGTAGCCA-3′. The 2^-ΔΔCT method was employed for data analysis [20].

### Acquisition and processing of single-cell sequencing data

The single-cell RNA sequencing (scRNA-seq) dataset GSE149614 was retrieved from the GEO database. The raw data, processed via the Cell Ranger pipeline, were integrated into Seurat objects (version 5.3.0) for downstream analysis. Stringent quality control (QC) was performed to retain high-quality cells based on the following criteria: 200 < gene counts < 5,000, UMI counts > 200, and mitochondrial gene content < 10%. Following the removal of low-quality cells and potential doublets, the data were log-normalized, and the top 2,000 highly variable genes (HVGs) were identified using the “vst” method. Batch effects across samples were corrected using the Harmony algorithm. Principal Component Analysis (PCA) was performed, and the top principal components (PCs) contributing to 80% of the cumulative variance were selected for dimensionality reduction. Cell clustering was performed at an optimal resolution, and the results were visualized using t-distributed Stochastic Neighbor Embedding (t-SNE). Cell identities were assigned to each cluster by cross-referencing highly differentially expressed genes with canonical lineage markers reported in previous liver cancer studies.

### Experimental validation and functional assays

The experimental protocols for Western blot (WB) and Cell Counting Kit-8 (CCK-8) are detailed in the Supplementary Methods.

### Statistical analysis

We utilized R (version 4.4.2) along with the required packages to conduct all statistical analyses in this study. *ZIC* expression levels in tumor and non-tumor tissues (NTs) were compared using the Wilcoxon rank sum test. Furthermore, relationships were evaluated using the Spearman correlation coefficient test. We utilized Kaplan–Meier curves in our survival analysis to evaluate the survival disparities between groups exhibiting high and low gene expression levels. For all survival analyses, patients were stratified into high- and low-expression groups based on the median mRNA expression level of the respective *ZIC* genes. Statistical significance was assessed using the log-rank test for each subgroup. A *p* < 0.05 was considered statistically significant. To control the false discovery rate (FDR) arising from multiple hypothesis testing across 33 cancer types, *p*-values were adjusted using the Benjamini-Hochberg (BH) procedure. An adjusted *p* < 0.05 was utilized to determine statistical significance.

## Results

### Expression of *ZIC* across cancers

**Fig. 1:**
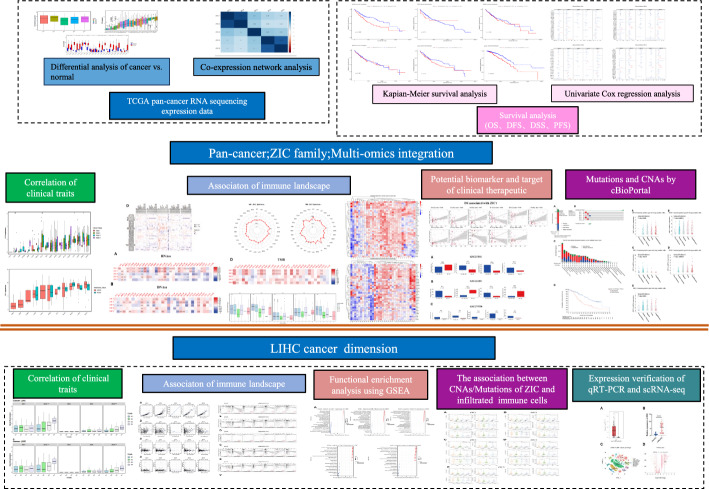
Flowchart illustrating the role of *ZIC* genes Credit: [All images created by the authors]

**Fig. 2:**
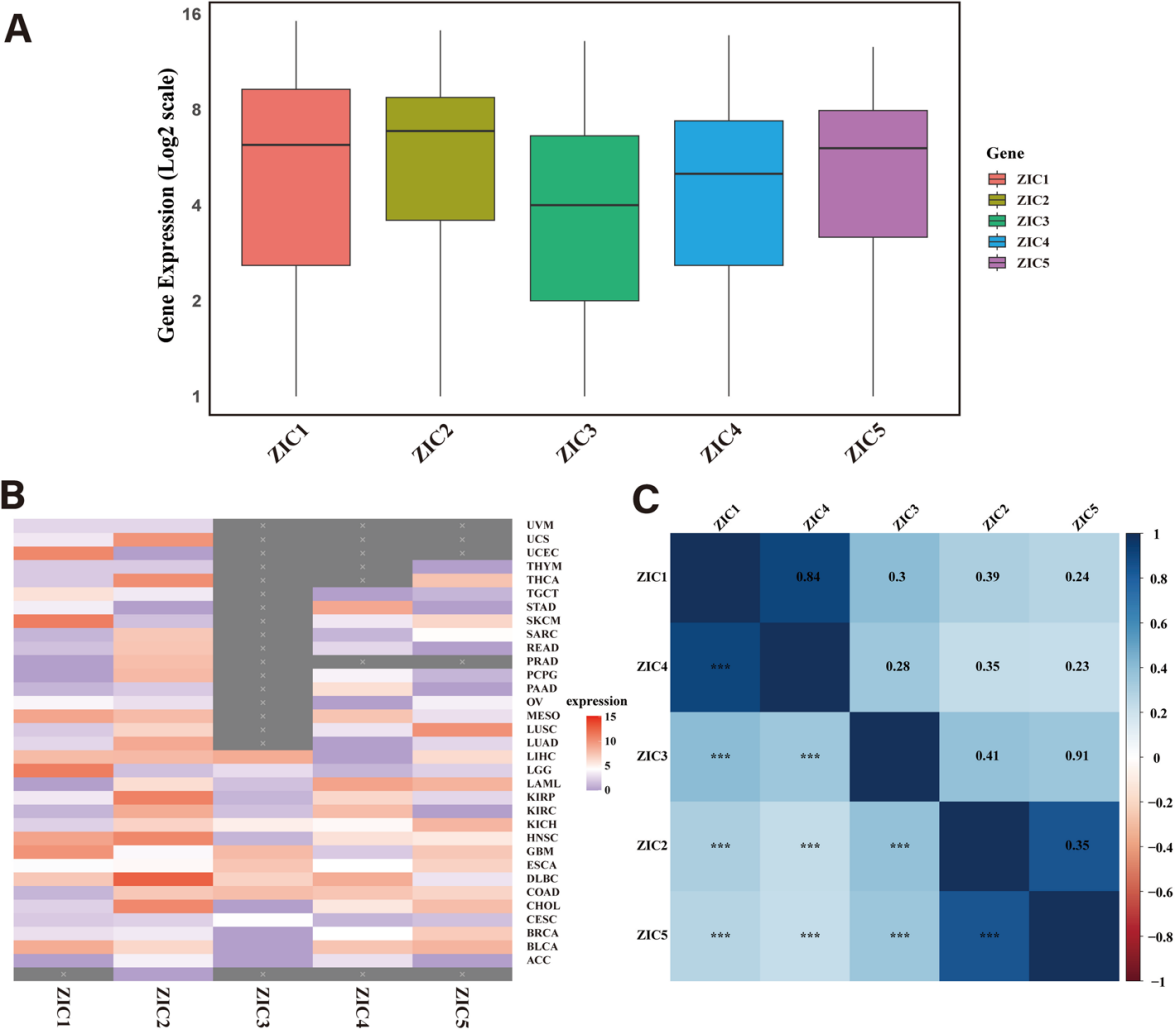
A thorough analysis of the expression variations and co-expression patterns of *ZIC* genes in pan-cancer. **A** A box plot showing the expression levels of *ZIC* genes in cancer tissues (CTs) derived from TCGA datasets. **B** Pan-cancer expression landscape of *ZIC* genes. Heatmap displays Z-score transformed mRNA levels (red: high; purple: low) in cancerous tissues and non-cancerous counterparts. Hierarchical clustering was performed using Euclidean distance and average linkage. Statistical significance was assessed by the Wilcoxon rank-sum test. **C** A correlation analysis among members of the *ZIC* gene family. ***p < 0.001. Exact *p*-values for all correlations are provided in supplementary table S4 Credit: [All images created by the authors]

Figure 1 outlines our comprehensive research workflow, beginning with pan-cancer expression analysis of five *ZIC* genes across 33 TCGA cancer types. Our findings revealed distinct expression patterns: mRNA levels of *ZIC1* and *ZIC2* were markedly elevated in most pan-cancer tissues (Fig. 2A), while *ZIC4* and *ZIC5* were moderately increased. In contrast, the expression levels of *ZIC3* remained relatively low across the majority of the analyzed cancer types (Supplementary Fig. S1A–E). Notably, all five *ZIC* genes exhibited consistently high expression in gliomas (GBM and LGG). Clinical correlations showed that elevated *ZIC1* and *ZIC5* expression is associated with poorer LGG survival, whereas reduced *ZIC3* levels predict better GBM outcomes [9]. These findings, along with the established role of *ZIC1* in medulloblastoma [23], underscore the significant involvement of *ZIC* family genes in neuro-oncological malignancies.

Specifically, Supplementary Fig. S1A highlights that *ZIC1* expression is exceptionally high in uterine carcinosarcoma (UCS) and sarcoma (SARC), as well as in gliomas (GBM and LGG), compared to other primary tumors. This study points to *TNNT1, COL1A2*, and *ZIC1* as potential biomarkers or molecular factors involved in driving the metastasis and proliferation of uterine sarcoma [24].

Compared with other cancer types, the *ZIC2* gene is significantly more highly expressed in esophageal carcinoma (ESCA) and CESC. ZIC2 collaborates with Gli1 to promote its retention in the nucleus, thereby amplifying the Hedgehog signaling pathway. The proliferation and migration of cervical cancer cells depend on this process [25]. However, research exploring *ZIC2* in the context of ESCA remains limited, positioning it as an underexplored candidate for future therapeutic advancements.

The expression of *ZIC3* is notably higher in testicular germ cell tumors (TGCT) and kidney chromophobe (KICH). Similarly, *ZIC4* and *ZIC5* expression is elevated in specific cancer types, including UCS, SARC, CESC, and ESCA. This is corroborated by the heatmap, which displays darker red hues for these tumors in contrast to the lighter shades observed across other cancer types (Fig. 2B).

Our analysis revealed distinct expression patterns of *ZIC* family genes through paired comparisons between tumor samples and matched adjacent normal tissues from the TCGA dataset (Supplementary Fig. S2B). *ZIC2* was consistently expressed at higher levels in the majority of cancers compared to their matched adjacent non-tumor tissues. In contrast, *ZIC1* demonstrated a significant reduction in cholangiocarcinoma (CHOL) and colon adenocarcinoma (COAD), while showing a marked increase in uterine corpus endometrial carcinoma (UCEC), along with further elevations in bladder urothelial carcinoma (BLCA) and head and neck squamous cell carcinoma (HNSC). In contrast, Supplementary Fig. S2E reveals that *ZIC5* showed predominantly lower levels in adjacent normal tissues across all the assessed cancers. Regarding *ZIC3*, its expression was significantly upregulated in LUAD, lung squamous cell carcinoma (LUSC), SARC, and KICH tissues compared with their respective matched normal controls (Supplementary Fig. S2C). Finally, *ZIC4* reached its maximum expression in GBM.

The correlations between pairs of *ZIC* family genes in pan-cancer tissues (PCTs) were examined using Spearman’s rank correlation coefficient. As shown in Fig. 2C, 10 gene pairs displayed positive associations. Specifically, *ZIC1* exhibited a robust correlation with *ZIC4* (r = 0.84), while *ZIC3* demonstrated a similarly strong correlation with *ZIC5* (r = 0.91).

### *ZIC* analysis in relation to pan-cancer expression, mutations, and copy-number (CN) changes

**Fig. 3:**
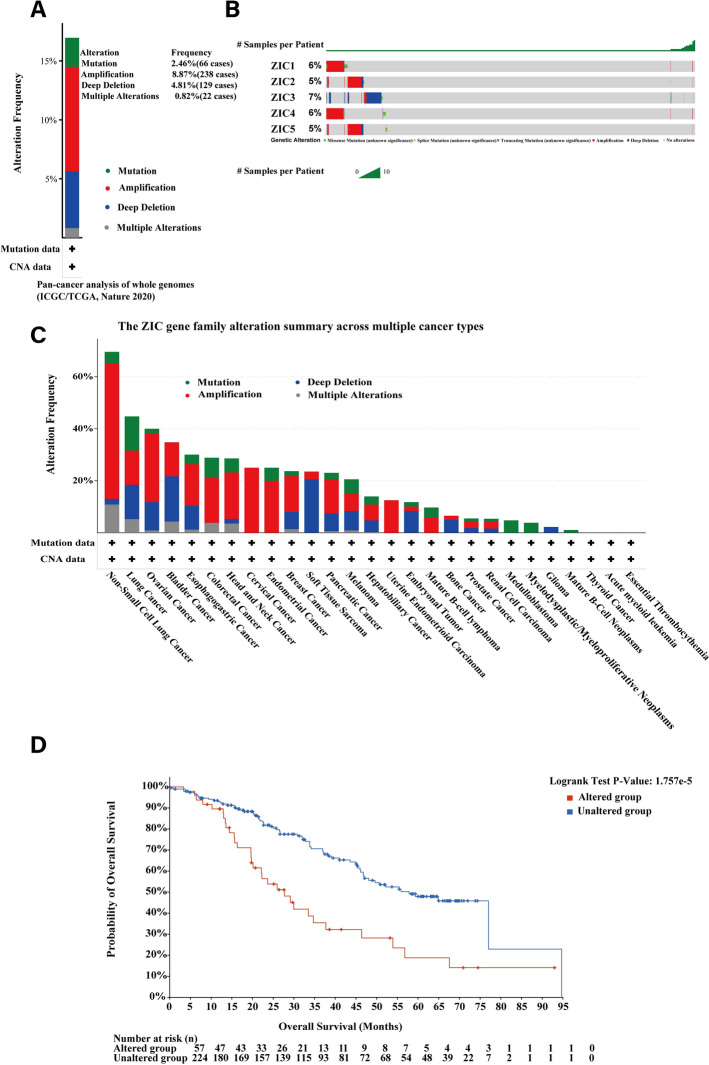
Analysis of *ZIC* Family Gene Alterations Across Pan-Cancer Using cBioPortal Data. **A** Overall frequency of *ZIC* alterations in pan-cancer. **B** An illustration of the chromosomal changes in *ZIC* genes. **C** *ZIC* alteration frequencies across various cancer types. **D** Kaplan–Meier survival analysis comparing overall survival in cases with versus without *ZIC* alterations Credit: [All images created by the authors]

We analyzed genetic alterations, including mutations, CNVs, and SNVs, in the *ZIC* gene family across multiple cancer types using the ICGC/TCGA dataset. In our study of *ZIC* genes across pan-cancer samples, we observed that approximately 17% of the samples (total *n* = 2,683 cases) exhibited genetic variations such as mutations, amplifications, and deep deletions (Fig. 3A). Among these, CNVs were the predominant alteration, impacting 389 cases (14.5%), far surpassing mutations, which affected only 66 cases (2.46%). Within the CNV category, amplifications emerged as the most common subtype, observed in 238 cases (8.87%). According to Fig. 3B, the proportion of genetic changes in *ZIC* genes fluctuated between 5 and 7% across different cancer types, with amplifications consistently being the leading alteration across all *ZIC* family members (except for *ZIC3*). Notably, *ZIC1* and *ZIC4* exhibited elevated amplification rates compared to *ZIC2* and *ZIC5*.

Figure 3C highlights unique genetic alteration profiles across 27 cancer types. Myelodysplastic neoplasms and mature B-cell neoplasms are chiefly characterized by mutations, whereas cervical cancer predominantly features amplifications. Deep deletions are a hallmark of glioma. For a detailed perspective, Supplementary Figure S3A-E outlines the frequency and spread of genetic changes across *ZIC* family members. Additionally, Fig. 3D presents a Kaplan–Meier (KM) survival analysis, revealing a markedly worse prognosis for patients with genetic alterations (p = 1.75 × 10^–5^).

**Fig. 4:**
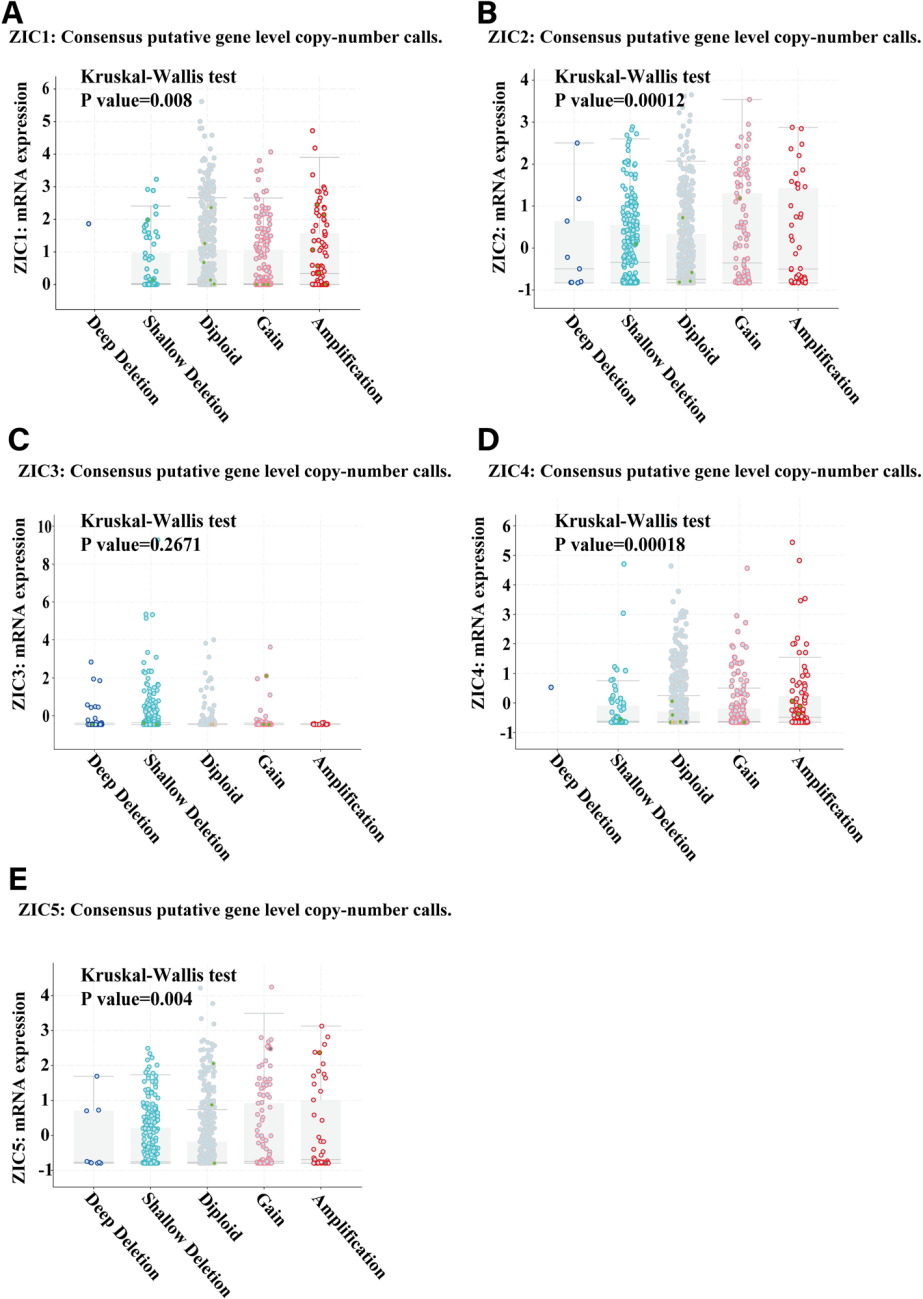
CNVs linked to particular *ZIC* family expression are investigated using cBioPortal’s ICGC/TCGA pan-cancer datasets (**A**–**E**) Credit: [All images created by the authors]

Furthermore, our analysis revealed an increased prevalence of genomic amplification within the pan-cancer cohort. In samples with increased copy numbers, the majority of *ZIC* genes exhibited significantly higher mRNA expression levels (Fig. 4). Significant disparities in *ZIC* mRNA expression were identified when comparing tumor samples with varying copy number profiles (deep deletions, shallow deletions, diploidy, gains, or amplifications). These observations suggest a potential influence of genomic copy number alterations on the transcriptional regulation of *ZIC* genes. The pan-cancer dataset contained more non-mutated areas than regions with missense, truncating, multiple, in-frame, or splice mutations, according to our analysis. Furthermore, *ZIC* mRNA expression levels did not significantly differ across the various genetic variation groups (Figure S4A–E). These results suggest that while the overexpression of *ZIC* mRNA in pan-cancer may be significantly associated with an increase in *ZIC* gene copy number, it is likely not the sole driver. Other regulatory mechanisms, such as epigenetic modifications (e.g., DNA methylation) or altered transcriptional factor activity, may also play critical roles in the transcriptional dysregulation of *ZIC* genes and warrant further mechanistic investigation.

### Analysis of relationships between TMB, MSI, stemness indices, and *ZIC* gene expression

**Fig. 5:**
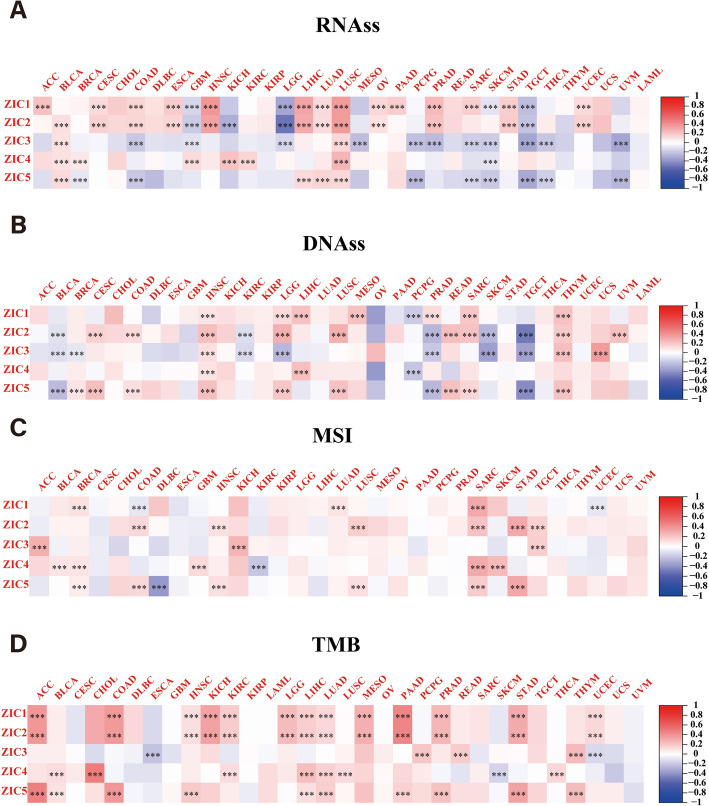
Correlation of *ZIC* family expression with stemness indices and genomic instability markers across the TCGA pan-cancer cohort. The heatmaps characterize the associations between *ZIC1*–*5* mRNA levels and several key oncogenic features: **A** RNAss, **B** DNAss, **C** MSI, and **D** TMB. The color gradient denotes Spearman’s rank correlation coefficient, where red and purple represent positive and negative correlations, respectively. Asterisks indicate statistical significance (***p < 0.001) Credit: [All images created by the authors]

Our findings revealed that the correlation (Spearman’s rank correlation) between RNAss and *ZIC* gene expression differed among tumors (Fig. 5A). Notably, in BLCA and LUSC, increased *ZIC* gene expression is associated with higher tumor stemness scores. We noticed a positive correlation between *ZIC* gene expression and DNAss in HNSC (Fig. 5B). Correlation analysis showed that *ZIC2* expression has a stronger correlation with tumor stemness indices than other *ZIC* genes.

Our investigation revealed a correlation between *ZIC* gene expression and MSI in various cancers (Fig. 5C). Specifically, a positive correlation was identified in SARC, BRCA, and STAD, while negative correlations emerged for KIRC and UCEC. In particular, *ZIC1* demonstrated a pronounced positive correlation with MSI in SARC, *ZIC2* in STAD, and *ZIC3* in ACC. These findings suggest that elevated *ZIC* gene expression may be associated with disrupted MMR system functionality.

As depicted in Fig. 5D, ZIC gene expression levels exhibit a higher frequency of significant correlations with TMB. Predominantly, *ZIC* gene expression shows a positive correlation with TMB. For example, in ESCA, increased *ZIC3* expression corresponds to a lower TMB. Strikingly, in the majority of tumors, fluctuations in *ZIC1* and *ZIC2* expression align positively with TMB. Additionally, higher *ZIC5* expression levels are associated with elevated TMB, which in turn is often linked to a more favorable response to immunotherapy. Supplementary Figures S5–S6 provide detailed correlation maps between *ZIC* family gene expression and both TMB and MSI across the 33 assessed cancer types. For example, Supplementary Fig. S5A confirms the robust correlation between *ZIC1* and TMB in ACC.

### Survival analysis of *ZIC* across cancers

**Fig. 6:**
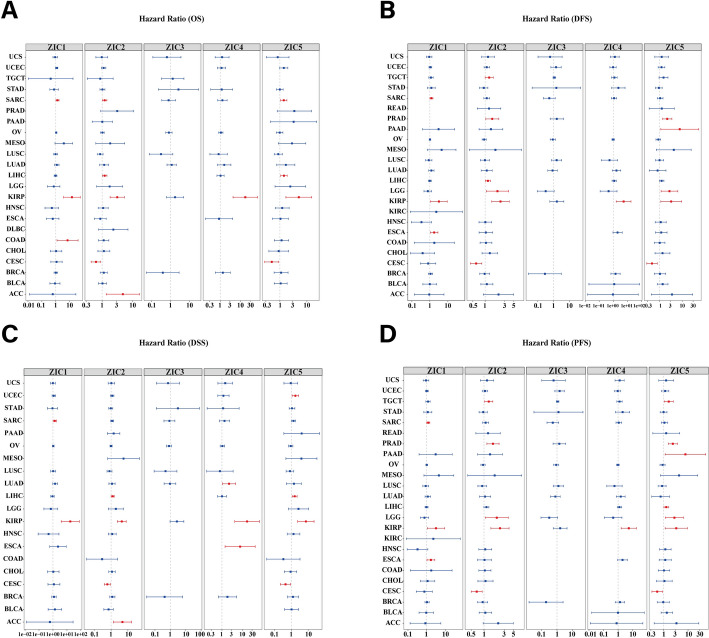
The connection between *ZIC* gene expression and clinical outcomes across various cancer types. **A** Overall survival (OS) among 23 distinct cancer types. **B** Disease-free survival (DFS) among 24 distinct cancer types. **C** Disease-specific survival (DSS) among 20 distinct cancer types. **D** Progression-free survival (PFS) among 24 distinct cancer types. The plots use distinct colors to indicate the statistical significance derived from the univariate Cox analysis: red highlights a significance level of *p* < 0.05, while blue denotes no statistically significant relationship Credit: [All images created by the authors]

Our pan-cancer analysis of TCGA data across 33 tumor types demonstrated significant prognostic associations between *ZIC* gene expression and clinical outcomes. KM survival analysis revealed strong correlations with OS, DFS, DSS, and PFS (the comprehensive survival metrics across 33 tumors are detailed in Supplementary Table S1). The results were corroborated by univariate Cox regression analysis, which established *ZIC* expression levels as significant predictors of OS (Fig. 6A), with consistent prognostic significance noted for DFS, PFS, and DSS (Fig. 6B–D).

Our analysis revealed distinct prognostic patterns for *ZIC* family members across various cancers. *ZIC1* demonstrated consistent high-risk associations in SARC and kidney renal papillary cell carcinoma (KIRP) across all survival endpoints (OS, DFS, DSS, PFS), with similar trends observed in ESCA for DFS and PFS. *ZIC2* emerged as a particularly strong prognostic marker in KIRP, showing significant correlations with poorer outcomes in OS, DFS, DSS and PFS, but displaying an opposite protective trend in CESC. Notably, *ZIC3* showed minimal prognostic relevance. *ZIC4* exhibited high-risk characteristics in KIRP, whereas *ZIC5* demonstrated significant risk associations in KIRP and LIHC (across three survival metrics, excluding DFS). These data highlight the potential of *ZIC* genes as clinically significant prognostic biomarkers, demonstrating especially robust predictive significance in KIRP across various family members.

### Effect of *ZIC* genes on pan-cancer staging and initial treatment response

Our research investigates the association between *ZIC* family gene expression and tumor pathological stages (reflecting the TNM classification system). We conducted a reanalysis of the TCGA database to investigate the dynamic changes in *ZIC* gene expression following the initial treatment of patients. Our pan-cancer analysis demonstrates that *ZIC* gene expression varies markedly across tumor types and their respective clinical stages (Fig. S7). For instance, Supplementary Fig. S7E highlights that *ZIC5* expression increases progressively with tumor stage in KIRP. Across 18 cancer types, *ZIC3* exhibited a markedly lower overall expression level relative to other *ZIC* family genes. In breast invasive carcinoma (BRCA) and TGCT, we found a weak association between *ZIC1* gene expression and disease stage (Fig. S7A). In contrast, in kidney renal clear cell carcinoma (KIRC), *ZIC2* expression levels increase progressively with tumor stage. Similarly, in TGCT, HNSC and LIHC, *ZIC2* expression tended to increase in the early stages but decreased in advanced stages (Fig. S7B). We revealed that *ZIC3* expression in COAD diminishes steadily with each clinical stage, while *ZIC4* expression in stage IV COAD was substantially higher than in stages I-III (Fig. S7C-D).

Following initial treatment, a notable link was observed between *ZIC* gene expression levels and therapeutic outcomes across various cancer types (Fig. S8). Post-treatment *ZIC* expression differed markedly depending on the cancer type. Our analysis reveals that *ZIC1* expression in patients with HNSC, LUAD, and LUSC is significantly lower in the CR/PR groups (Fig. S8A).

*ZIC2*, expression was elevated in the PD/SD groups of adrenocortical carcinoma (ACC) and HNSC patients, potentially suggesting a connection with unfavorable treatment responses (Fig. S8B). The expression of *ZIC3* showed distinct trends: it was reduced in the CR/PR groups for ACC and HNSC, yet elevated in the CR/PR group for LUSC (Fig. S8C). In BLCA, *ZIC4* expression was diminished in the PD/SD groups, suggesting that higher levels might confer a protective effect (Fig. S8D). Moreover, *ZIC5* expression was increased in the PD/SD groups of ACC and LUAD (Fig. S8E). In summary, after initial therapy, elevated *ZIC4* expression appears to be a beneficial factor associated with improved clinical outcomes, whereas high levels of *ZIC1* and *ZIC2* may indicate a poorer prognosis.

### *ZIC*'s function: an investigation involving many different IT cohorts

**Fig. 7:**
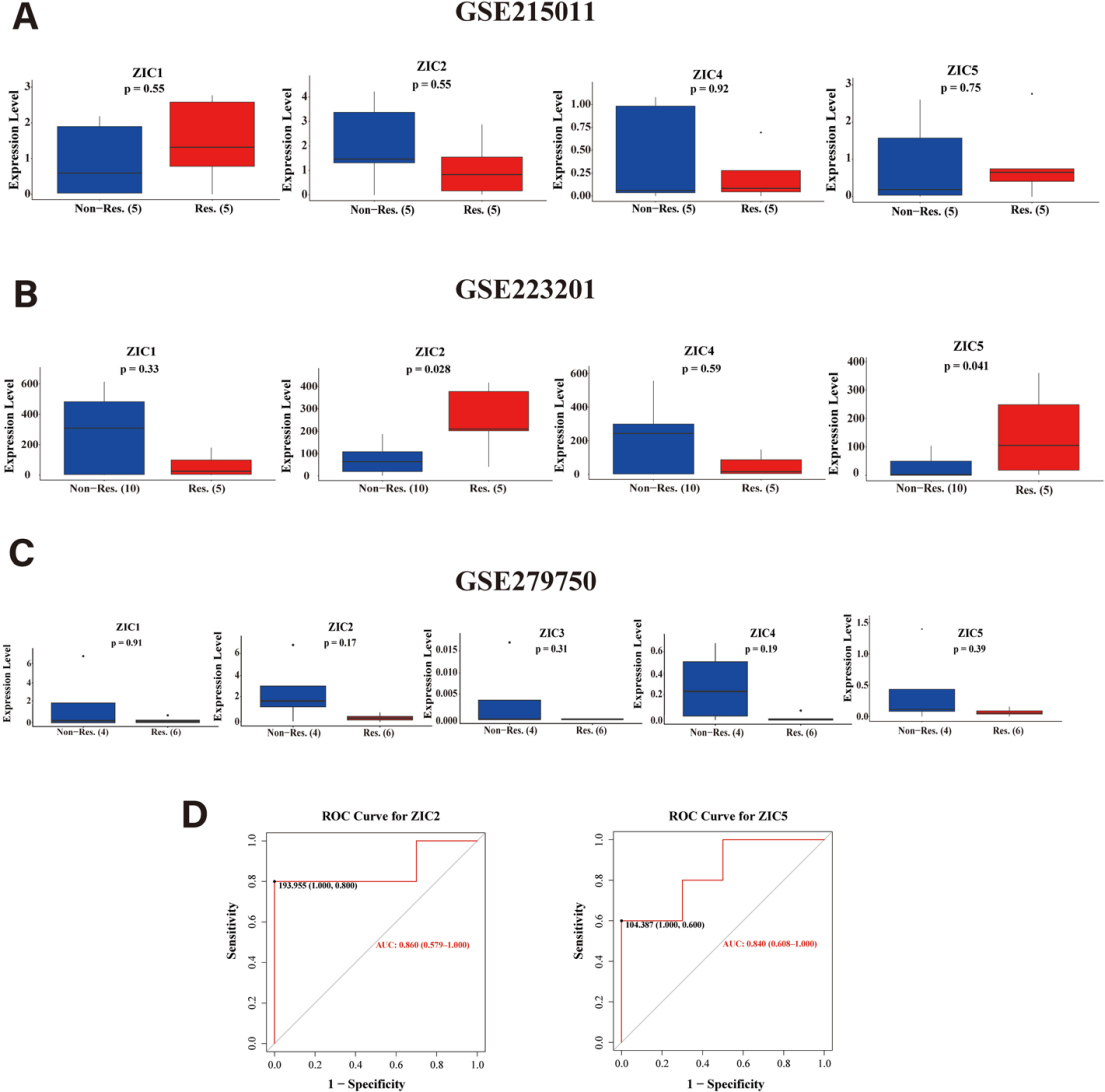
The expression levels of the *ZIC* family were investigated across several datasets, namely GSE215011, GSE223201, and GSE279750. **A** In the GSE215011 dataset, *ZIC* expression was analyzed across distinct groups classified by their response status to immunotherapy. **B** In the GSE223201 dataset, alterations in *ZIC* expression levels were observed before and after immunotherapy. **C** In the GSE279750 dataset, alterations in *ZIC* expression levels were observed before and after immunotherapy Credit: [All images created by the authors]

Given the clinical importance of IT, we performed an exploratory analysis of the association between *ZIC* genes and IT outcomes across three small cohorts (GSE215011, GSE223201, and GSE279750). As illustrated in Fig. 7A, in the GSE215011 and GSE279750 cohorts, *ZIC* gene expression in liver cancer patients exhibited no notable correlation with IT efficacy. In contrast, within the GSE223201 cohort (*n* = 15), elevated *ZIC2* and *ZIC5* expression levels were associated with improved IT responsiveness. Given the extremely limited sample sizes of these cohorts (*n* = 10 for GSE215011, *n* = 15 for GSE223201, and *n* = 10 for GSE279750), these findings are preliminary and exploratory in nature. The observed discrepancies—where significant correlations for *ZIC2* and *ZIC5* were found only in the GSE223201 cohort—underscore the potential lack of statistical power and the high heterogeneity across small clinical datasets. Therefore, these results should be interpreted with significant caution and serve primarily as a rationale for future large-scale prospective validation.

In the GSE223201 cohort, we further assessed the exploratory predictive potential of *ZIC* genes using ROC curve analysis and univariate logistic regression. As illustrated in Fig. 7D, *ZIC2* and *ZIC5* exhibited preliminary discriminatory power between responders and non-responders, with *AUC* values of 0.860 (95% *CI*: 0.579–1.000) and 0.840 (95% *CI*: 0.608–1.000), respectively. Univariate logistic regression revealed a positive association trend between gene expression and therapeutic response (*ZIC2*: *OR* = 1.017, 95% CI 1.004–1.043; *ZIC5*: *OR* = 1.016, 95% CI 1.002–1.044, Supplementary Table S1). Although the limited sample size (*n* = 15) and the wide confidence intervals suggest a potential risk of statistical overfitting, these exploratory *AUC* metrics and positive *OR* values provide preliminary evidence for of *ZIC2* and *ZIC5* as potential candidate biomarkers for predicting IT efficacy in liver cancer.

### Exploring the interplay of *ZIC* gene expression, tumor immune subtypes, and TME

**Fig. 8:**
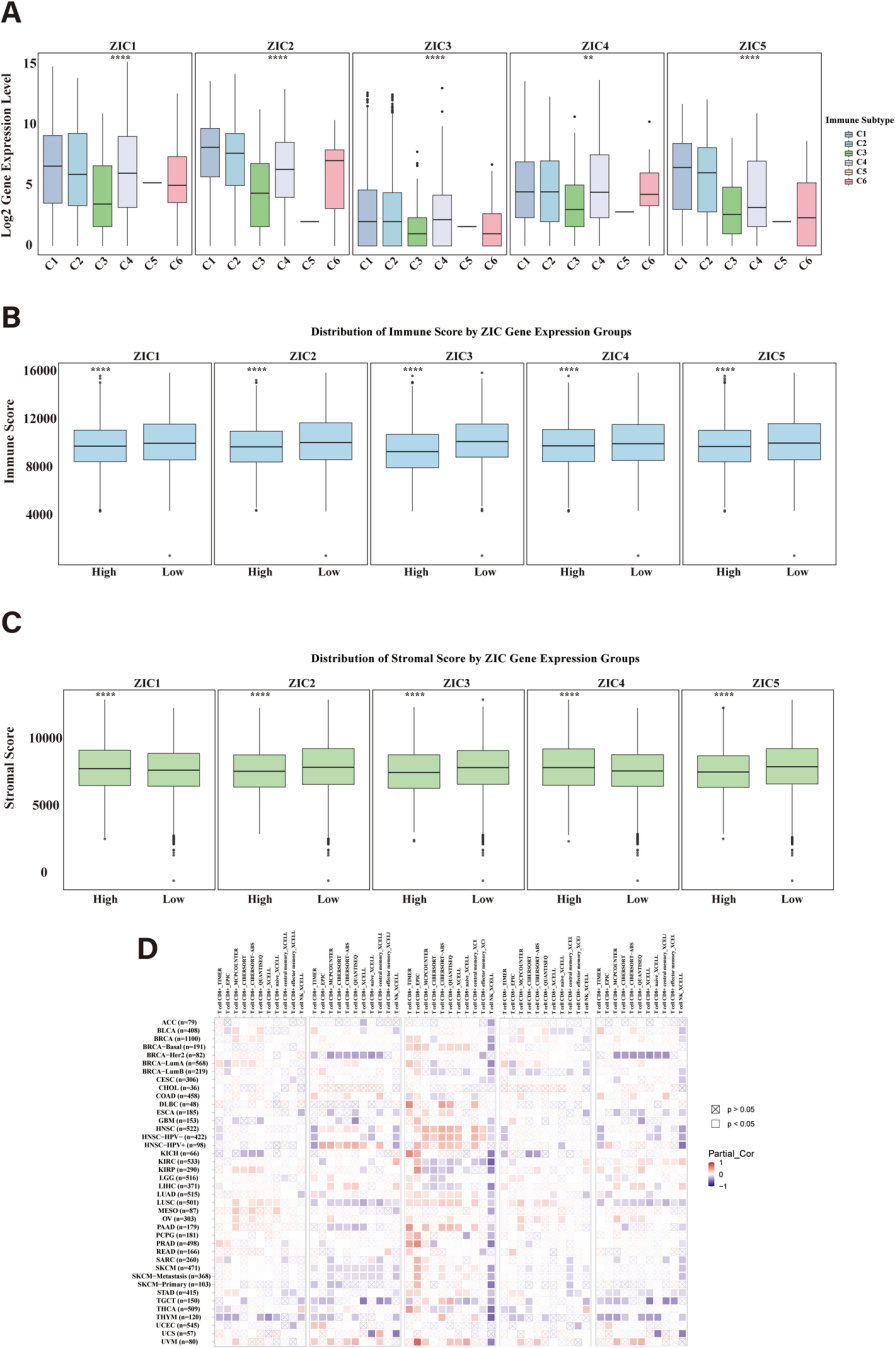
The connections between *ZIC* family expression and TCGA pan-cancer datasets' immune subtypes, immunological scores, stromal scores, and immune cell infiltration. **A** Analysis of the relationship between *ZIC* family expression and immune subtypes using TCGA data. **B**, **C** Relationships between *ZIC* expression and immune/stromal scores. **D** Correlation analysis between *ZIC* family expression and immune cell infiltration. The heatmap displays Spearman's rank correlation coefficients between *ZIC1*–*5* expression and the infiltration levels of CTLs, NK cells, and NKT cells. Infiltration abundance was estimated using various deconvolution algorithms. The color gradient represents the correlation coefficient, with red indicating a positive correlation and purple indicating a negative correlation. Asterisks signify statistical significance. **p < 0.01, ****p < 0.0001 Credit: [All images created by the authors]

Figure 8A reveals that *ZIC* family genes exhibit specific expression signatures across the six immune subtypes (C1–C6). In PCTs, *ZIC1* and *ZIC2* display consistently elevated expression levels across all subtypes, suggesting a complex role in tumor immunity. Notably, *ZIC1* achieves its highest expression in the C1 subtype, whereas *ZIC2* peaks in C1 and C2. Significant relationships (evaluated via Spearman’s rank correlation) between ESTIMATE scores and *ZIC* family gene expression were found (Fig. 8B, C). Specifically, immune scores exhibited an inverse relationship with the expression of *ZIC1*–*ZIC5* (Fig. 8B). Lower *ZIC* gene expression was associated with elevated immune scores, suggesting that reduced *ZIC* expression may facilitate greater immune cell presence in the TME. A parallel trend was observed for stromal scores (Fig. 8C), where higher expression of *ZIC1* and *ZIC4* corresponded to increased stromal scores.

We examined the link between *ZIC* gene expression and immune cell infiltration (Fig. 8D). In PCTs, *ZIC3* expression demonstrated a significant positive association with immune cell infiltration, surpassing that of other *ZIC* family members; however, this pattern was inverted for NKT cells. Across all five *ZIC* genes, a consistent negative correlation with NKT cell levels was observed. In LUSC, *ZIC2* expression exhibits a negative correlation with infiltration, while in HNSC-HPV + , it displays a positive correlation, except for CD8 + T cells and NKT cells. These results highlight the intricate and cancer-type-specific associations of *ZIC* family genes in shaping the tumor immune microenvironment.

### Co-expression analysis of ***ZIC*** genes and immune checkpoint genes (ICGs)

Tumor ICGs are pivotal in the context of IT. We assessed 38 ICGs to investigate their link to *ZIC* gene expression using Spearman’s rank correlation analysis (Fig. S9 A-E). As shown in Supplementary Fig. S9A, *ZIC1* expression significantly correlates with most ICGs in GBM and LGG. Our findings revealed that, for *ZIC1*, the majority of ICGs displayed positive correlations with expression levels in PCTs. Specifically, in GBM, LGG, and BRCA, *ZIC1* expression exhibited a significant positive association with ICG expression. Intriguingly, in CHOL and UCS, elevated *ZIC1* expression was linked to reduced ICG levels, potentially suggesting diminished immune suppression or lower responsiveness to specific immunotherapies.

For *ZIC2*, positive correlations with ICG expression were observed across most tumor types. In contrast, *ZIC3* expression showed predominantly negative correlations with ICGs across various tumors, particularly in KIRC, and TGCT. Moreover, *ZIC4* expression closely aligned with ICG levels in UVM, KIRC, and BRCA. In several cancer types, including LGG, and GBM, there was a positive correlation between *ZIC5* expression and the ICGs. Conversely, the opposite pattern was observed for LUSC, and SARC. These results suggest that *ZIC* family genes may participate in the regulation of the immune checkpoint landscape.

### Evaluation of DS in relation to *ZIC* gene expression

Using the CellMiner database based on the NCI-60 cell line panel, we conducted a comprehensive analysis to investigate the potential pharmacological significance of *ZIC* expression. Our findings revealed significant correlations between *ZIC* levels and sensitivity to several clinically established anticancer agents and mechanistically relevant compounds.

**Fig. 9:**
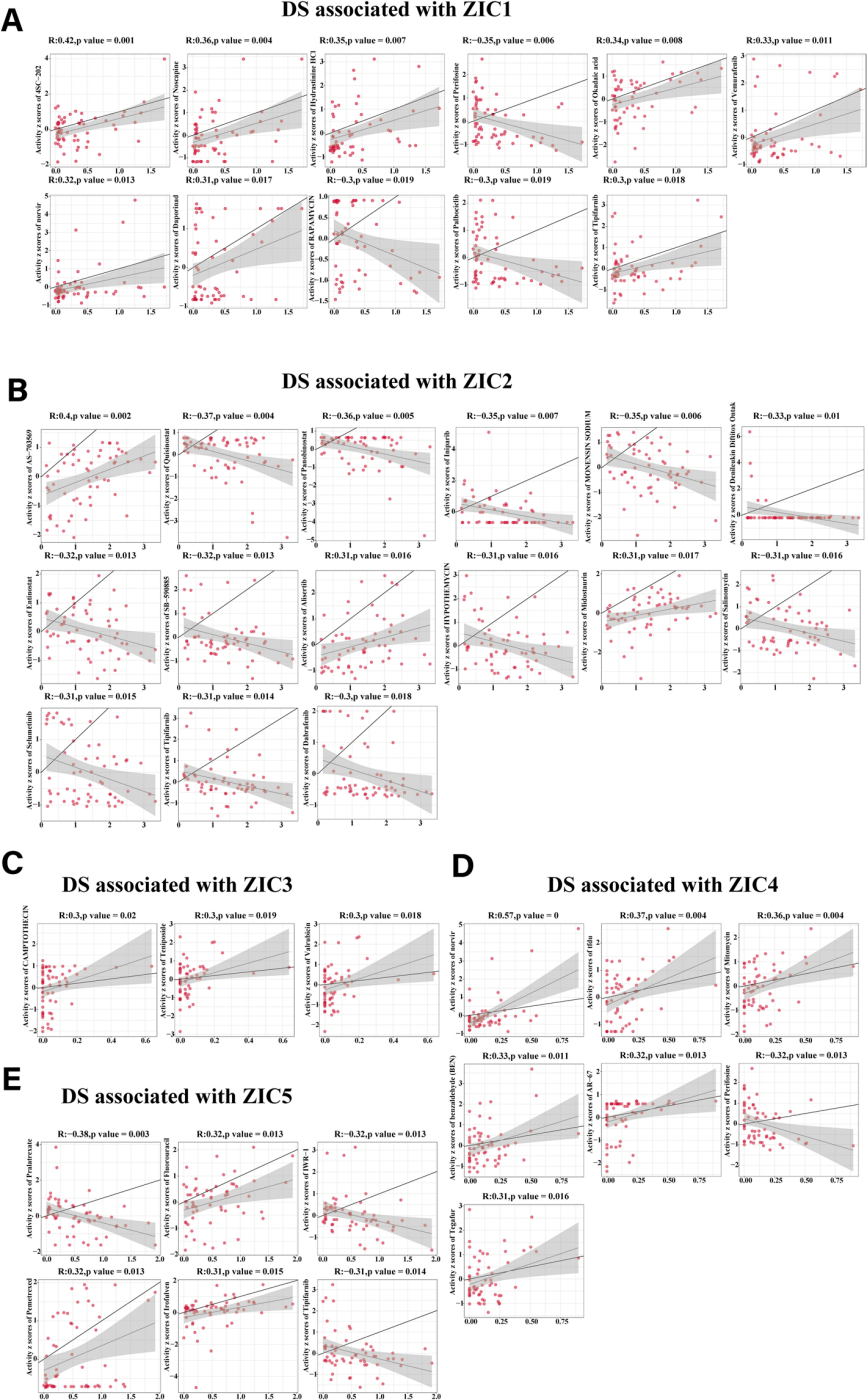
Analysis of medication reactions linked to variations in *ZIC* exp. in various TCGA malignancies. **A** Notable relationships between *ZIC1* expression levels and 11 drugs (*p* < 0.05). **B** Key associations between *ZIC2* expression and 15 drugs. **C** Significant relationships between *ZIC3* expression levels and 3 drugs. **D** Significant relationships between *ZIC4* expression levels and 7 drugs. **E** Key associations between *ZIC5* gene expression levels and 6 drugs. Raw *p*-values are shown in the figures. The corresponding FDR adjusted *p*-values, which account for multiple hypothesis testing, are provided in Supplementary Table S2 Credit: [All images created by the authors]

For *ZIC1*, higher expression was positively associated with increased sensitivity to the BRAF inhibitor vemurafenib (Fig. 9A), while an inverse relationship was observed for the mTOR inhibitor rapamycin and the CDK4/6 inhibitor palbociclib. In the case of *ZIC2*, although DS predominantly showed a negative correlation with expression (Fig. 9B), most notably, sensitivity to alisertib and AS-703569 was significantly positively correlated with *ZIC2* expression.

Similarly, elevated *ZIC3* expression was linked to increased sensitivity to DNA-damaging agents such as camptothecin, valrubicin and teniposide (Fig. 9C). For *ZIC4* and *ZIC5*, we observed that higher expression predicted better sensitivity to several compounds, including Norvir and fluorouracil (Fig. 9D, E). In contrast, sensitivity to signaling modulators like perifosine and IWR-1 exhibited opposite trends. These results indicate that *ZIC* family genes, particularly *ZIC2* and *ZIC5*, may serve as potential indicators for the efficacy of specific kinase inhibitors and antimetabolite chemotherapies. Detailed data on all statistically significant drug correlations are available in Supplementary File S2. Notably, the correlation between *ZIC4* and Norvir remained significant after Benjamini–Hochberg correction (FDR = 0.019), while other candidates showed higher FDR values due to the stringent statistical penalty of high-throughput screening in the small NCI-60 panel (*n* = 60).

### Enrichment analysis of *ZIC* gene expression in LIHC

While pan-cancer analysis highlighted multiple malignancies, LIHC was prioritized due to the robust mRNA upregulation of *ZIC2/5* in paired tissues and the unique prognostic impact of *ZIC5* on OS, DSS, and PFS. Furthermore, liver cancer-specific cohorts identified *ZIC2/5* as potential exploratory immunotherapy predictors (*AUC* > 0.84). This multifaceted evidence establishes LIHC as the optimal model for translating bioinformatic findings into clinical insights.

**Fig. 10:**
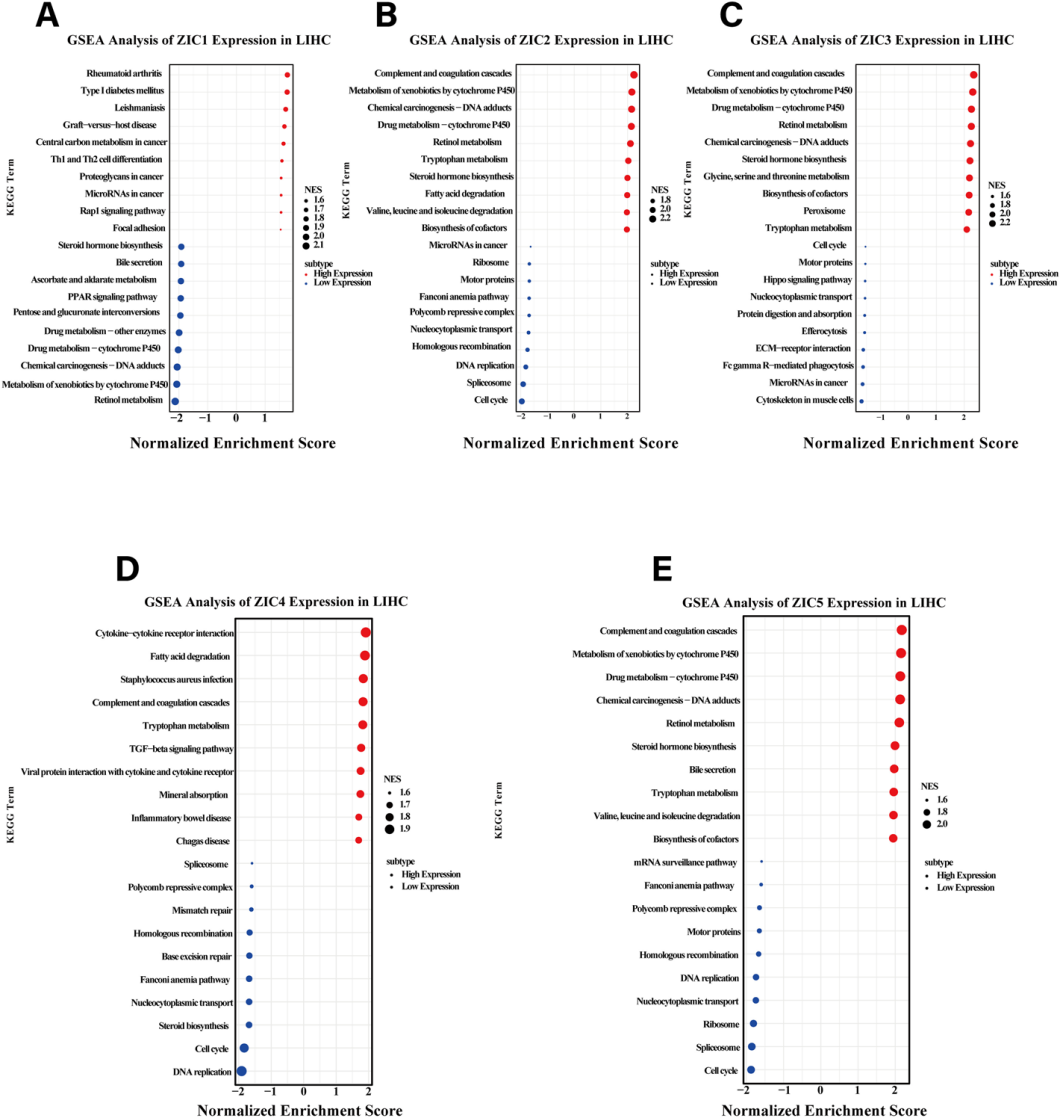
Individual *ZIC* family members were subjected to GSEA **A**–**E** Credit: [All images created by the authors]

GSEA identified significant pathway divergence between *ZIC* expression groups in LIHC, encompassing cell cycle regulation, DNA replication, and immune-related signaling (Fig. 10). Specifically, high *ZIC2* expression was associated with the activation of multiple processes, including complement cascades and cytochrome P450-mediated processing, while cell cycle, spliceosome, and DNA replication pathways were notably downregulated. This distinct functional landscape highlights the intricate regulatory role of *ZIC2* in LIHC, prompting our subsequent investigation into its tumor-intrinsic biological contributions to hepatocellular oncogenesis.

### Association between *ZIC* expression and immune subtypes and clinicopathological features in LIHC

**Fig. 11:**
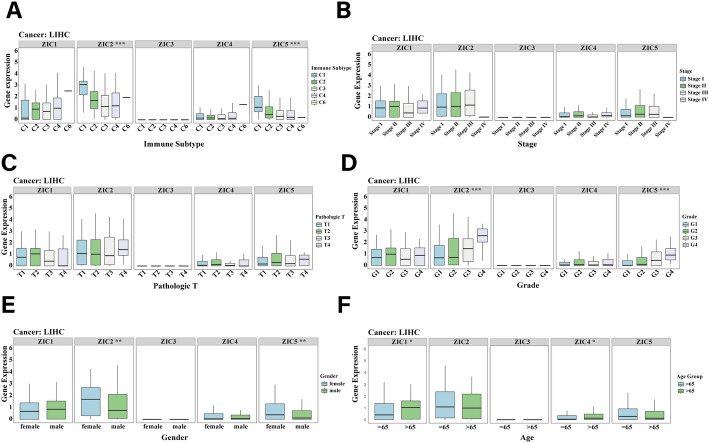
Correlations between clinicopathological characteristics and *ZIC* expression. **A** *ZIC* expression levels by immunological subtype. **B**–**F** Box plots showing *ZIC* expression levels in association to immunological subtypes, tumor stage, pathologic T stage, histological grade, age, and gender, among other clinicopathological features in LIHC. **p < 0.01, ***p < 0.001, *p < 0.05 Credit: [All images created by the authors]

We found that the expression of most *ZIC* genes varies significantly across the C1-C4 and C6 immune subtypes. Specifically, *ZIC2* demonstrated the highest expression across all subtypes except C6, with both *ZIC2* and *ZIC5* exhibiting significantly elevated levels in the C1 subtype compared with other subtypes (Fig. 11A). This pattern suggests that elevated *ZIC* expression in liver cancer is associated with specific immune microenvironment characteristics.

Our study examined how *ZIC* expression relates to clinicopathological characteristics in LIHC patients (Fig. 11B–F). While the expression levels of *ZIC1*–*ZIC5* showed no statistically significant association with clinical stage or pathologic T, further analysis revealed that higher expression of *ZIC2* and *ZIC5* is significantly associated with more advanced tumor grades (G3–G4). Additionally, female LIHC patients displayed higher *ZIC2* and *ZIC5* expression than male patients. Regarding age, individuals over 65 years exhibited increased *ZIC1* and *ZIC4* expression compared to those under 65 years. These findings suggest that, in liver cancer, elevated *ZIC2* expression—particularly in the C1 immune subtype and high-grade tumors—may correlate with more aggressive tumor characteristics.

### Analysis of *ZIC* expression in relation to stemness, stromal score, TMB, and MSI in LIHC

**Fig.12:**
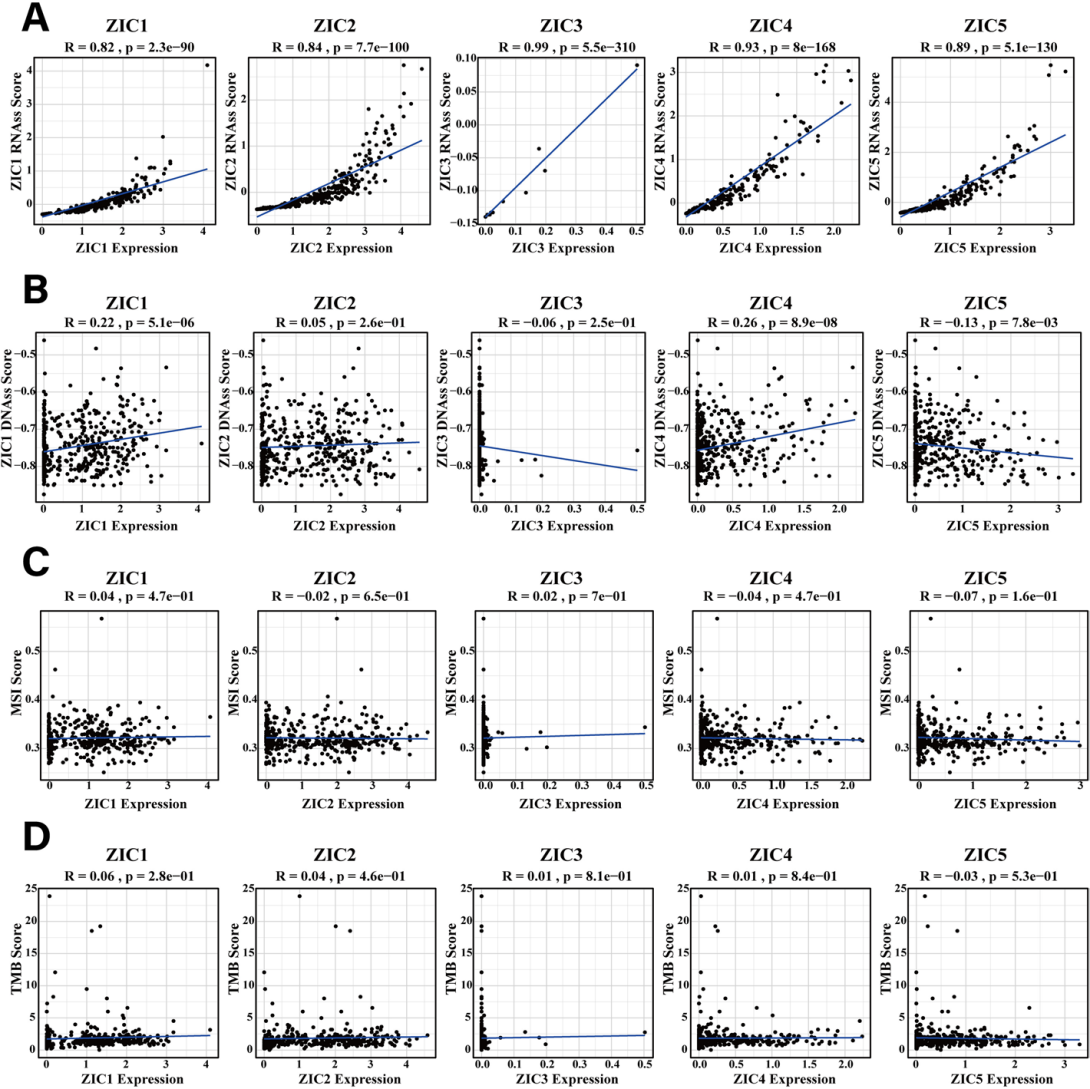
Connections between *ZIC* expression and key features in LIHC. **A** Correlations between *ZIC* expression and RNAss. **B** Relationships between *ZIC* expression and DNAss. **C** Associations between *ZIC* expression and MSI. **D** Interactions between *ZIC* expression and TMB Credit: [All images created by the authors]

Our analysis demonstrated a statistically significant positive correlation (*p* < 0.05) between *ZIC1*—5 expression and the RNA stemness index in LIHC (Fig. 12A–D). Conversely, in relation to the DNAss, *ZIC1* and *ZIC4* displayed a weak positive association, whereas *ZIC5* exhibited a minor negative association. These findings indicate that *ZIC* genes associated with elevated stemness indices may contribute to tumor progression, metastasis, and resistance to treatment in LIHC. Further analysis exploring the association between *ZIC* expression and MSI or TMB identified no significant relationships.

### Correlation between *ZIC* expression and TME in LIHC

**Fig. 13:**
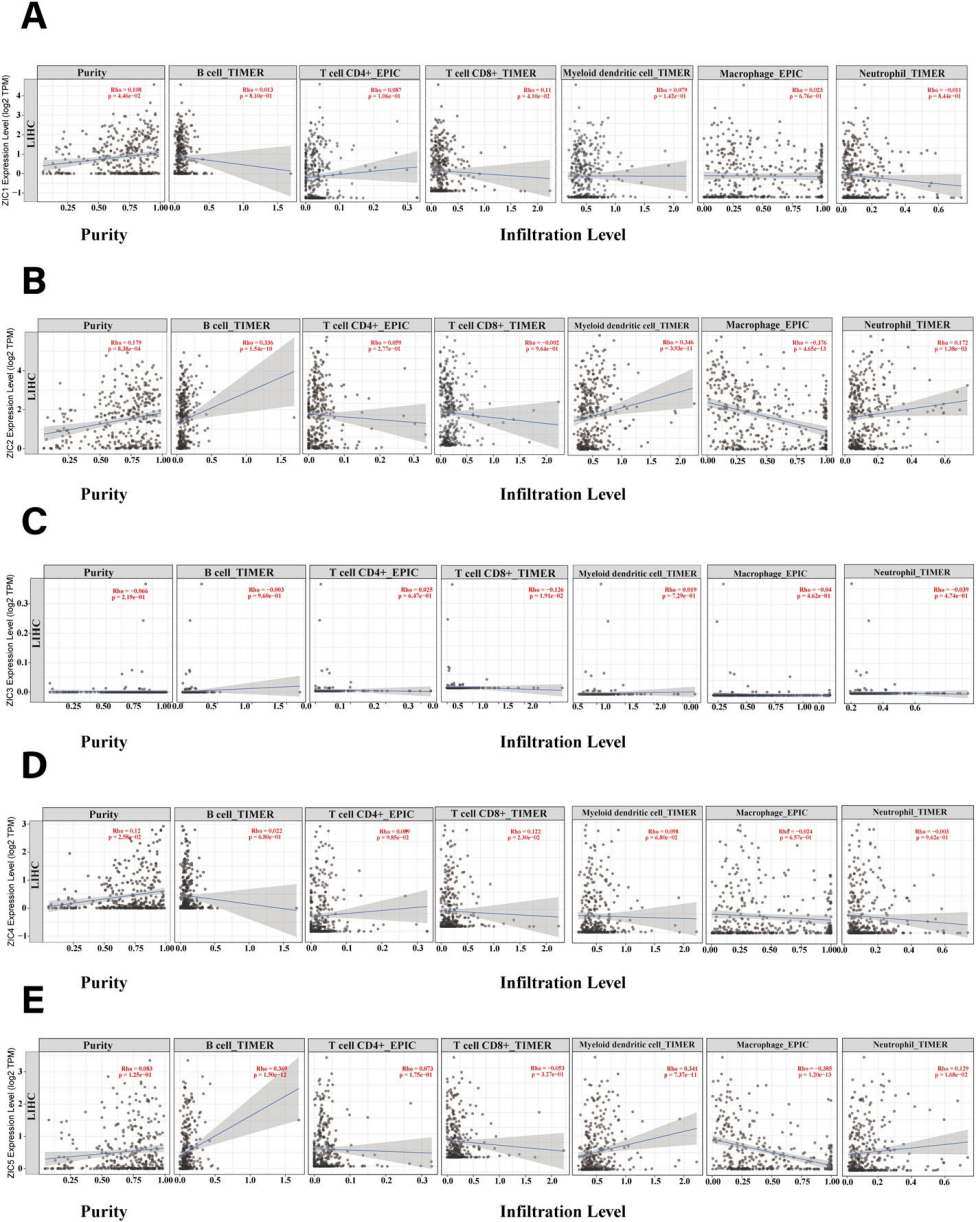
Associations between *ZIC* expression and immune cell infiltration levels in LIHC **A**–**E** Credit: [All images created by the authors]

Furthermore, our study investigated the relationship between *ZIC* gene expression and the abundance of six key immune cell populations in TME of LIHC. As shown in Fig. 13, the expression of *ZIC1* displayed a modest positive association with CTL infiltration, but exhibited negligible connections with B cells, Ths, MΦs, PMNs, and DCs. In contrast, *ZIC2* expression was positively associated with B cells and DCs, while showing an inverse association with macrophages. *ZIC3* expression, however, showed little to no link with any of the six major immune cell populations. For *ZIC4*, a faint positive connection was observed with CTL infiltration. Moreover, *ZIC5* expression demonstrates a substantial positive correlation with B cells and DCs (R > 0.3), alongside a pronounced negative correlation with macrophages. Our findings suggest that *ZIC* gene expression may be linked to the LIHC immune landscape, potentially reflecting the complex interplay between tumor cells and the immune microenvironment.

### Correlation between *ZIC* gene CNVs and immune infiltration in LIHC

**Fig. 14:**
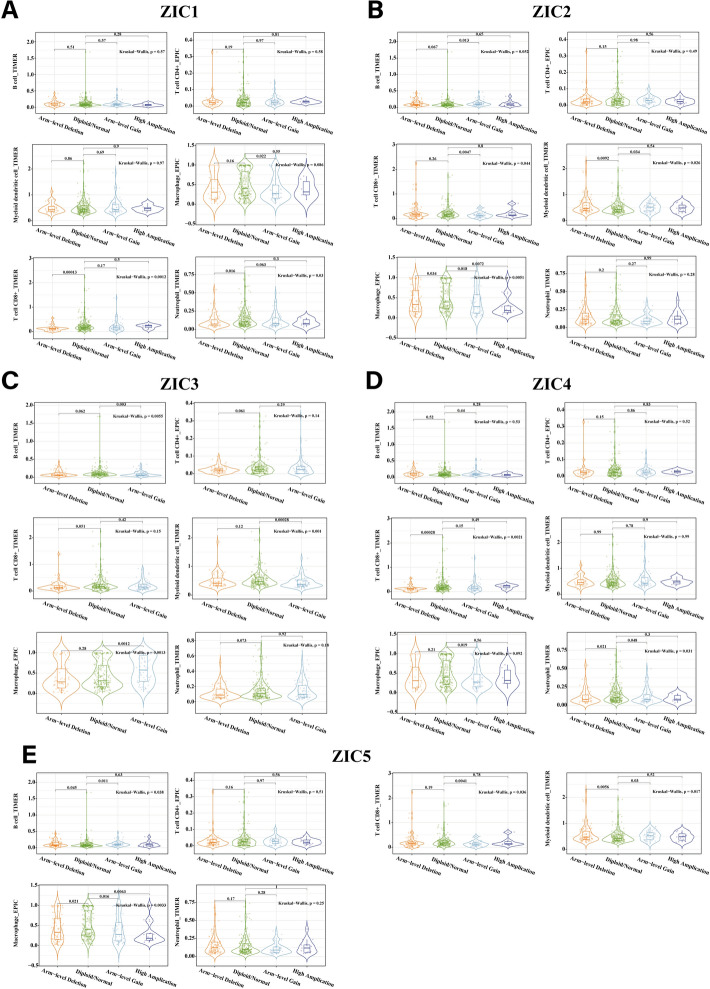
*ZIC* CNV mutations and immunological infiltrates in LIHC are related. **A** CNVs of *ZIC1* gene in LIHC. **B** CNVs of *ZIC2* gene in LIHC. **C** CNVs of *ZIC3* gene in LIHC. **D** CNVs of *ZIC4* gene in LIHC. **E** CNVs of *ZIC5* gene in LIHC Credit: [All images created by the authors]

The association between immune cell infiltration in LIHC and CNVs in *ZIC* genes was analyzed. For *ZIC1*, CNVs exhibited a significant association with infiltration levels of CTLs and PMNs (Kruskal–Wallis, *p* < 0.05). Notably, arm-level deletions were associated with reduced infiltration compared to diploid samples (Fig. 14A). In *ZIC2*, CNVs significantly correlated with the infiltration of CTLs, DCs, and MΦs (Kruskal–Wallis, *p* < 0.05). Arm-level deletions correlated with decreased CTLs and MΦs infiltration, whereas arm-level gains were linked to elevated DC infiltration (Fig. 14B). For *ZIC3*, arm-level gains corresponded to reduced B-cell and DC infiltration, contrasted by heightened MΦs infiltration (Fig. 14C). Similarly, in *ZIC4*, arm-level deletions were associated with diminished CTLs and PMNs infiltration (Fig. 14D). Similarly, *ZIC5* exhibited a consistent trend in CTLs and MΦs, whereas its infiltration level was elevated in DCs (Fig. 14E).

### Confirmation of *ZIC2* gene expression in LIHC

**Fig. 15:**
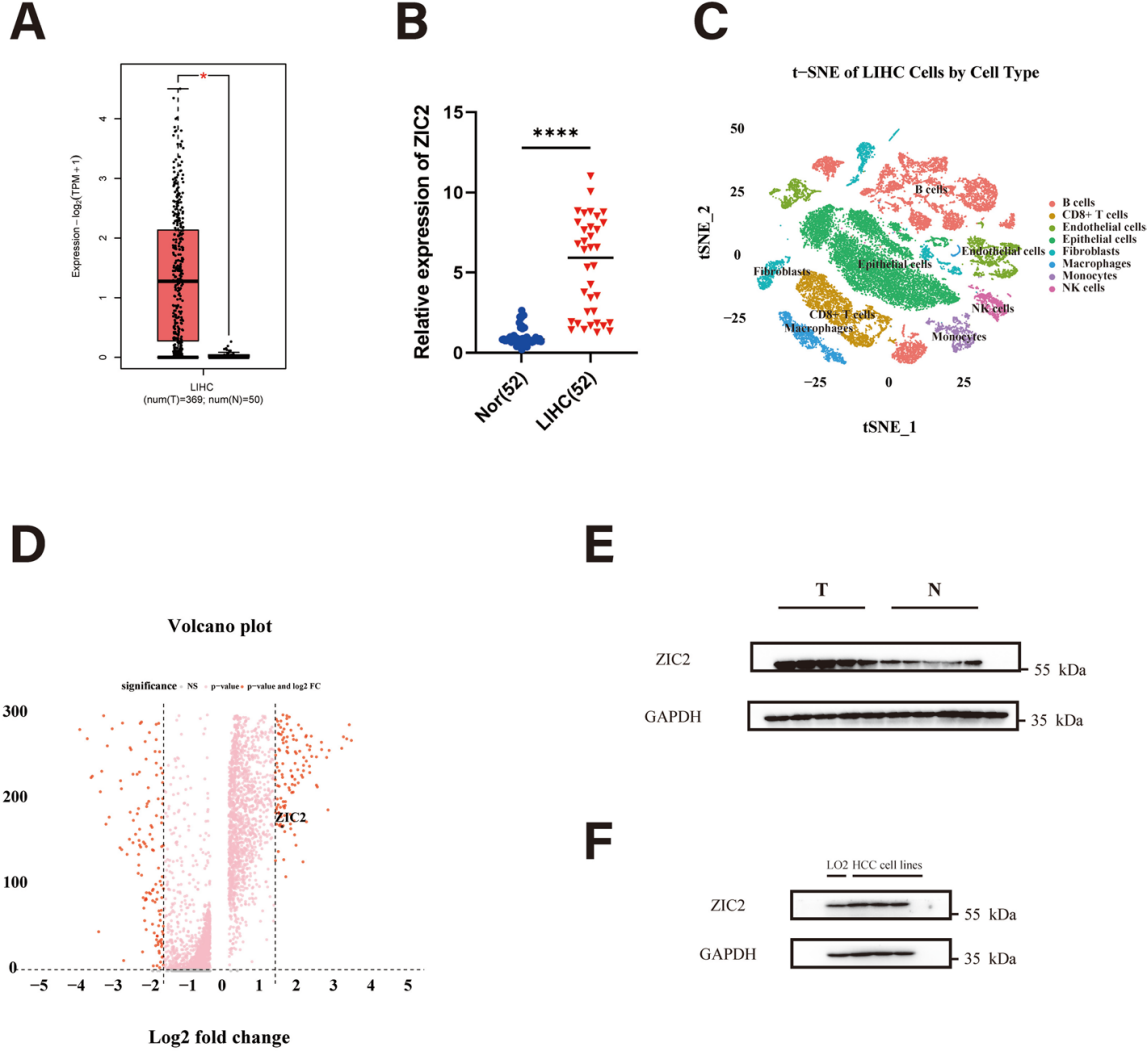
Validation of *ZIC2* mRNA and scRNA-seq in Clinical Samples. **A** *ZIC2* mRNA expression in LIHC and normal liver tissues. **B** *ZIC2* mRNA expression level in 52 pairs of LIHC with adjacent tissue are shown by the qRT-PCR data. **C** The distribution of cell types was visualized. **D** Volcanic map of differential genes. **E** *ZIC2* protein expression in 5 pairs of LIHC tumor (T) and matched adjacent normal (N) tissues. **F** *ZIC2* protein expression in the normal liver cell line (LO2) and HCC cell lines (HepG2, HuH-7, and Hep3B). *p < 0.05, ****, *p* < 0.0001 Credit: [All images created by the authors]

To further validate these findings using an unpaired comparison with pooled normal samples, analysis of the GEPIA2 database disclosed significantly elevated *ZIC2* expression in LIHC tumor tissues (Fig. 15A). To experimentally validate these observations, we performed qRT-PCR analysis on 52 paired LIHC clinical samples, which confirmed the significant upregulation of *ZIC2* in tumor tissues (Fig. 15B; Supplementary Table S3).

To further characterize the cellular landscape of LIHC, single-cell RNA sequencing data (GSE149614) were investigated. After stringent quality control and filtering (Supplementary Fig. S10A–B), a total of 71,915 cells were retained for subsequent analysis. Using t-SNE dimensionality reduction and clustering, the resulting plot (Fig. 15C) identified eight distinct cell categories. These clusters were systematically annotated based on the expression of canonical marker genes: B cells (e.g., *CD79A*, *MS4A1*), CD8 + T cells (e.g., *CD8B*, *GZMK*), endothelial cells (e.g., *VWF*, *PECAM1*), epithelial cells/hepatocytes (e.g., *ALB*, *KRT8*), fibroblasts (e.g., *COL1A1*, *FAP*), macrophages (e.g., *CD68*, *CD163*), monocytes (e.g., *CD14*, *FCN1*), and NK cells (e.g., *KLRB1*, *NKG7*). This high-resolution mapping of 71,915 cells provides a robust foundation for examining the cell-type-specific expression of *ZIC2* within the liver TME. Differential gene expression analysis (Tumor cells vs. Normal cells) was visualized in a volcano plot (Fig. 15D), confirming that *ZIC2* is prominently and significantly upregulated in the tumor cells (*p_adj* < 0.05). Given that the epithelial cell/hepatocyte cluster (Fig. 15C) constitutes the primary malignant cell population in the LIHC samples, these results strongly suggest that the elevated *ZIC2* signal is tumor-intrinsic. This provides a robust foundation for interpreting its correlation with the immune microenvironment as being mediated by tumor-derived signaling. To further corroborate the transcriptomic findings at the protein level, Western blot analysis was conducted. ZIC2 protein expression was markedly elevated in five pairs of LIHC tumor tissues compared to their matched adjacent normal tissues (Fig. 15E). Consistently, significantly higher ZIC2 protein levels were observed in HCC cell lines (HepG2, HuH-7, and Hep3B) relative to the normal liver cell line LO2 (Fig. 15F), providing multi-scale evidence for ZIC2 upregulation in LIHC. Consistently, CCK-8 assays revealed that these high-ZIC2 HCC cell lines exhibited significantly greater sensitivity to Alisertib compared to LO2 cells (Supplementary Fig. S9F), validating the therapeutic vulnerability predicted by our bioinformatic analysis.

## Discussion

The *ZIC* family of transcription factors (*ZIC1*-*ZIC5*) plays a pivotal function in cancer biology, extending its influence beyond neural development to the tumorigenesis of various cancer types. While previous studies have concentrated on individual *ZIC* genes in specific cancers, this research marks the first comprehensive pan-cancer evaluation of the entire *ZIC* family. Leveraging multi-omics data from the TCGA alongside validation cohorts, we investigated their expression profiles, prognostic value, genomic alterations, immune interactions, and therapeutic implications, following the established framework for systematic pan-cancer biomarker discovery [26, 27]. Our findings demonstrate the crucial role of *ZIC* genes in LIHC and their dual functions as tumor suppressors or oncogenes, depending on the cancer type.

The expression of *ZIC1*-*ZIC5* varies widely across 33 cancer types, with pronounced upregulation observed in GBM, LGG, UCS, and SARC. These results are in line with previous research, such as the link between *ZIC1* and *ZIC5* and reduced survival in glioma patients [6], as well as *ZIC2*’s association with aggressive behavior in CESC and ESCA [21]. In LIHC, *ZIC2* and *ZIC5* are markedly elevated, correlating with higher tumor grades and distinct immune subtypes, suggesting a role in driving cancer progression.

Prognostic assessments underscore the clinical importance of *ZIC* genes. Elevated *ZIC1* expression emerged as a risk factor in SARC and KIRP, however, high expression of *ZIC2* was also associated with poor prognosis in KIRP and LIHC. In contrast, *ZIC2* and *ZIC5* expression demonstrated a protective association in CESC, suggesting a favorable prognostic correlation that underscores their context-specific roles. It is important to note that while these findings identify *ZIC2* and *ZIC5* as favorable prognostic markers in the CESC cohort, these represent statistical associations rather than proven tumor-suppressive functions, necessitating future experimental validation to elucidate their biological roles in this specific cancer type. These observations align with *ZIC2*’s oncogenic role in colorectal cancer through the TGF-β pathway [9], while its potential as a tumor suppressor in other settings merits further investigation.

Across the pan-cancer cohort, genomic alterations—chiefly CNVs—were prevalent, with amplifications being the dominant subtype (8.87%). These CNVs strongly correlated with heightened *ZIC* mRNA levels, a pattern corroborated by ICGC/TCGA data. In LIHC, CNVs in *ZIC2* and *ZIC5* are associated with shifts in immune infiltration, indicating that gene dosage may amplify expression and shape tumor biology. Survival analysis revealed poorer outcomes in patients with *ZIC* alterations (*p* = 0.0000175), underscoring the prognostic value of these genomic changes. The direct downstream targets and chromatin-level mechanisms of *ZIC* genes warrant further investigation via ChIP-seq or ATAC-seq.

A central insight from this study is the intricate interplay between *ZIC* gene expression and the TME, underscoring the importance of high-resolution TME mapping through integrative multi-omics and single-cell sequencing [28]. *ZIC* expression was tied to immune subtypes (C1-C6), immune scores, and stromal scores (computed via the ESTIMATE algorithm) across multiple cancers. In LIHC, *ZIC2* and *ZIC5* showed robust positive associations with B-cell and dendritic cell infiltration, but a negative correlation with macrophages, suggesting their regulatory influence on immune dynamics. This finding aligns with previous reports on the impact of *ZIC2* immune infiltration in lung adenocarcinoma [16]. To reconcile these diverse associations, we propose that *ZIC* genes act as context-dependent molecular rheostats of the TME. In “immune-cold” tumors like LGG, *ZIC* signaling may support immunosuppression by maintaining stemness; this is consistent with evidence that the crosstalk between programmed cell death (PCD) and cancer stem cell (CSC) characteristics is a pivotal regulator of the TME [29]. conversely, in “hot” tumors like LIHC, high *ZIC* expression (driven by CNV) may coincide with genomic instability, thereby exhibiting a strong positive correlation with the infiltration B cells and dendritic cells. Notably, this framework reconciles the prognostic-predictive paradox observed in LIHC. While high *ZIC2* expression indicates clinical aggressiveness and poor overall survival, its association with an immunologically active TME, which may reflect a potentially pro-inflammatory phenotype often characterized as a “cold-to-hot” transition [30], renders these high-risk tumors more susceptible to checkpoint blockade. This framework suggests that the immunological impact of *ZIC* family members is governed by tissue-specific transcriptional programs and pathway crosstalk, such as Wnt/β-catenin signaling.

Analysis of DS indicated that *ZIC* expression levels could predict responses to anticancer therapies. High *ZIC1* expression was associated with sensitivity to BRAF inhibitor such as vemurafenib, whereas *ZIC2* expression was correlated with responsiveness to kinase inhibitors such as alisertib. These insights position *ZIC* genes as candidate biomarkers for tailored treatment; specifically, *ZIC1* and *ZIC2* expression could serve as companion diagnostics to guide the selection of BRAF and kinase inhibitors, providing concrete scenarios for precision oncology. While most drug associations yielded FDR > 0.05 (reflecting the exploratory nature of CellMiner screenings), the identification of significant hits like Norvir and the experimental validation of Alisertib highlight the biological relevance of these prioritized candidates.

In immunotherapy, elevated *ZIC2* and *ZIC5* expression in the GSE223201 LIHC cohort was related to greater reactivity to anti-PD-1/PD-L1 therapy, highlighting their predictive value. This finding resonates with the increasing focus on biomarkers for immunotherapy success [6]. The enrichment of immune pathways in LIHC further bolsters the possibility of therapeutically targeting *ZIC*-mediated immune regulation. Given their significant upregulation and prognostic link, integrating *ZIC2* and *ZIC5* into clinical scoring systems could enhance early-stage risk stratification and post-operative monitoring in LIHC. However, it must be noted that our exploration across multiple immunotherapy cohorts yielded mixed results. The extremely small sample sizes across the available GSE datasets (*n* = 10–15) significantly limit the generalizability of our claims and introduce a severe risk of statistical overfitting, as evidenced by the wide 95% confidence intervals in our ROC analysis. Such inter-cohort contradictions, characterized by significant correlations were observed in GSE223201 but not in others, may stem from differences in treatment regimens, patient backgrounds, and the inherent statistical fragility of small-n studies. We emphasize that these findings are preliminary and exploratory, intended to provide a theoretical and hypothesis-generating basis for the role of *ZIC* genes in immunotherapy, rather than serving as a definitive clinical tool at this stage. Validation in large-scale, prospective clinical trials is mandatory to confirm these preliminary observations.

While broad in scope, this study has several limitations. First, the dependence on public datasets may introduce potential selection bias. Second, while ZIC2 upregulation in LIHC was validated via qRT-PCR and Western blot, the absence of extensive in vivo models, direct in vitro co-culture assays and pan-cancer functional characterization restricts our ability to establish definitive causality. Consequently, the potential role of *ZIC* genes in shaping the TME remains a transcriptomic-based hypothesis that warrants future mechanistic validation. Thirdly, as this study is primarily based on bioinformatics analysis of public datasets, the observed relationships between *ZIC* expression and tumor features (such as immune infiltration and drug sensitivity) are correlational rather than causal. Fourthly, it must be acknowledged that the NCI-60 panel used for drug sensitivity screening lacks HCC lines. While our CCK-8 assays provided independent validation for Alisertib in HCC lines, the initial pan-cancer extrapolation may still introduce tissue-specific bias. Therefore, pharmacological predictions for LIHC derived from this study should be interpreted with caution. While these associations provide valuable insights into the pan-cancer landscape of the *ZIC* family, further mechanistic experiments are required to elucidate the specific biological pathways involved.

Future efforts should focus on functional experiments to elucidate the molecular mechanisms behind *ZIC* genes’ context-dependent roles, especially in LIHC and GBM, where their prognostic and therapeutic relevance stand out. Exploring downstream targets and pathways, including hedgehog, Wnt/β-catenin, and TGF-β, previously tied to *ZIC* function will enrich our understanding [9]. Additionally, examining *ZIC* interactions with the immune system could unlock new immunotherapeutic avenues.

## Supplementary Information

Additional file 1.

Additional file 2.

Additional file 3.

Additional file 4.

Additional file 5.

Additional file 6.

Additional file 7.

Additional file 8.

Additional file 9.

Additional file 10.

Additional file 11.

Additional file12

Additional file 13.

Additional file 14.

Additional file 15.

Additional file 16.

Additional file 17.

Additional file 18.

Additional file 19.

Additional file 20.

Additional file 21.

Additional file 22.

## Data Availability

The datasets generated and/or analyzed during the current study are available in the following repositories: 1. Public transcriptomic and single-cell datasets were retrieved from the Gene Expression Omnibus (GEO) repository (https://www.ncbi.nlm.nih.gov/geo/) under accession numbers GSE215011, GSE223201, GSE279750, and GSE149614. 2. Pan-cancer multi-omics data, including gene expression and clinical phenotypes, were obtained from The Cancer Genome Atlas (TCGA) via the UCSC Xena platform (https://xenabrowser.net/datapages/). 3. Genomic alteration and mutation data were analyzed through the cBioPortal for Cancer Genomics (https://www.cbioportal.org/). 4. Immune infiltration and tumor microenvironment analysis were supported by data from the TIMER2.0 (Tumor Immune Estimation Resource) website (http://timer.comp-genomics.org/). 5. Drug sensitivity profiles were accessed from the CellMiner database (https://discover.nci.nih.gov/cellminer/). 6. Gene expression validation across various cancers was performed using the GEPIA2 (Gene Expression Profiling Interactive Analysis 2.0) website (http://gepia2.cancer-pku.cn/). 7. The experimental validation data (including qRT-PCR results and original Western blot images) generated during the current study are provided in Supplementary Table S3.

## Abbreviations

TCGA: The Cancer Genome Atlas
RNA: Ribonucleic acid
OS: Overall survival
DFS: Disease-free survival
DSS: Disease-specific survival
PFS: Progression-free survival
CR: Complete response
PR: Partial response
PD: Progressive disease
SD: Stable disease
TMB: Tumor mutation burden
MSI: Microsatellite instability
cBioPortal: CBio Cancer Genomics Portal
GSEA: Gene set enrichment analysis
DNAss: DNA methylation-based stemness index
RNAss: RNA-based stemness index
FC: Fold change
ACC: Adrenocortical carcinoma
BLCA: Bladder urothelial carcinoma
BRCA: Breast invasive carcinoma
CESC: Cervical squamous cell carcinoma and endocervical adenocarcinoma
CHOL: Cholangiocarcinoma
COAD: Colon adenocarcinoma
DLBC: Diffuse large B-cell lymphoma
ESCA: Esophageal carcinoma
GBM: Glioblastoma multiforme
HNSC: Head and neck squamous cell carcinoma
KICH: Kidney chromophobe
KIRC: Kidney renal clear cell carcinoma
KIRP: Kidney renal papillary cell carcinoma
LAML: Acute myeloid leukemia
LGG: Brain lower grade glioma
LIHC: Liver hepatocellular carcinoma
LUAD: Lung adenocarcinoma
LUSC: Lung squamous cell carcinoma
MESO: Mesothelioma
OV: Ovarian serous cystadenocarcinoma
PAAD: Pancreatic adenocarcinoma
PCPG: Pheochromocytoma and paraganglioma
PRAD: Prostate adenocarcinoma
READ: Rectum adenocarcinoma
SARC: Sarcoma
SKCM: Skin cutaneous melanoma
STAD: Stomach adenocarcinoma
TGCT: Testicular germ cell tumors
THCA: Thyroid carcinoma
THYM: Thymoma
UCEC: Uterine corpus endometrial carcinoma
UCS: Uterine carcinosarcoma
UVM: Uveal melanoma
DS: Drug sensitivity
ICGs: Immune checkpoint genes
