# Supplementary figures and images for: Multi-omics integration reveals pan-cancer roles of ZIC family genes in prognosis, immune microenvironment remodeling, and therapeutic vulnerability
Source: Discov Oncol. 2026 Apr 28;17:890. doi: 10.1007/s12672-026-05100-2 (PMC13253929; doi:10.1007/s12672-026-05100-2)

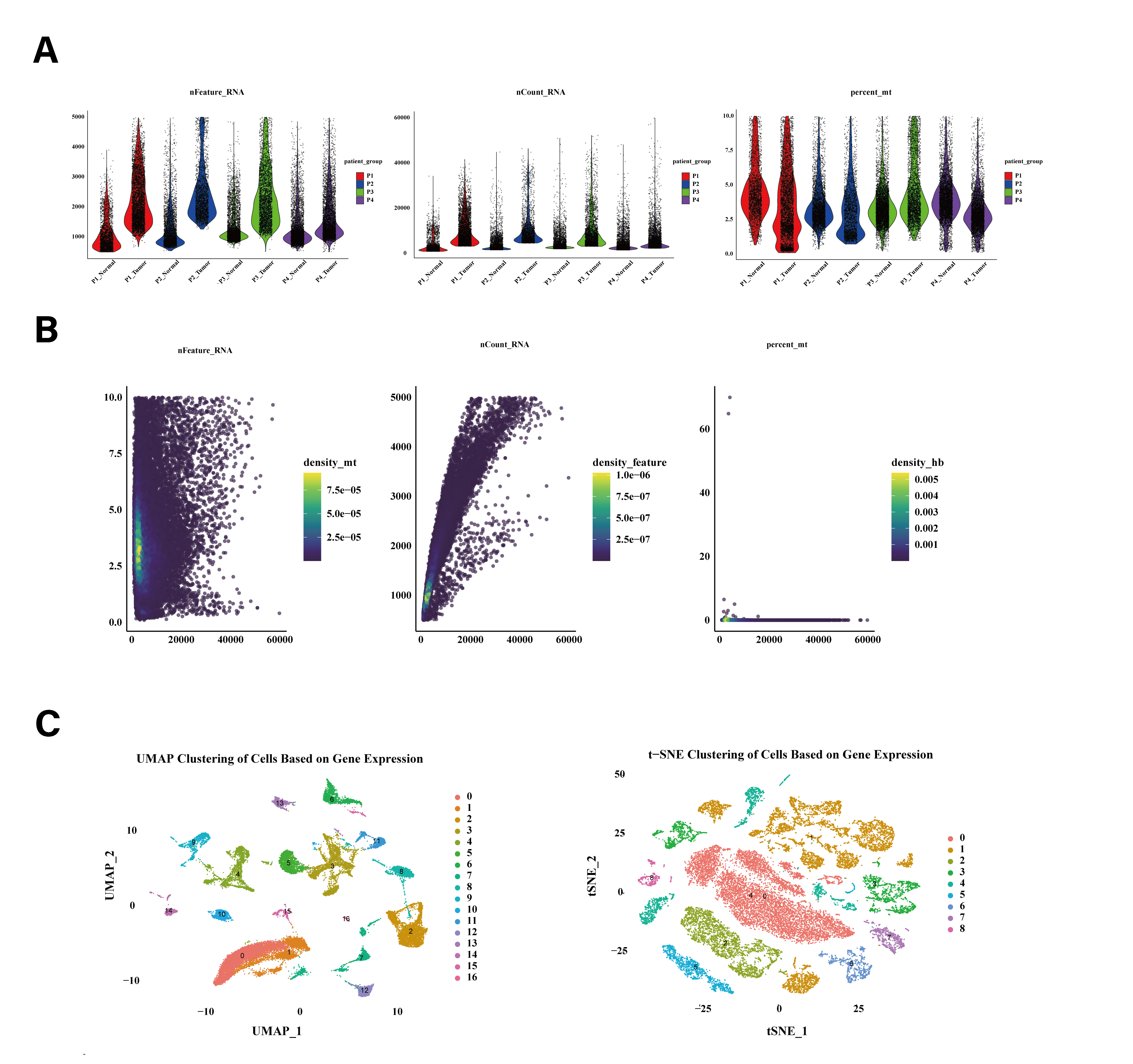

Supplement: Supplementary file 2 — Additional file 2. [file 12672_2026_5100_MOESM2_ESM.tif]

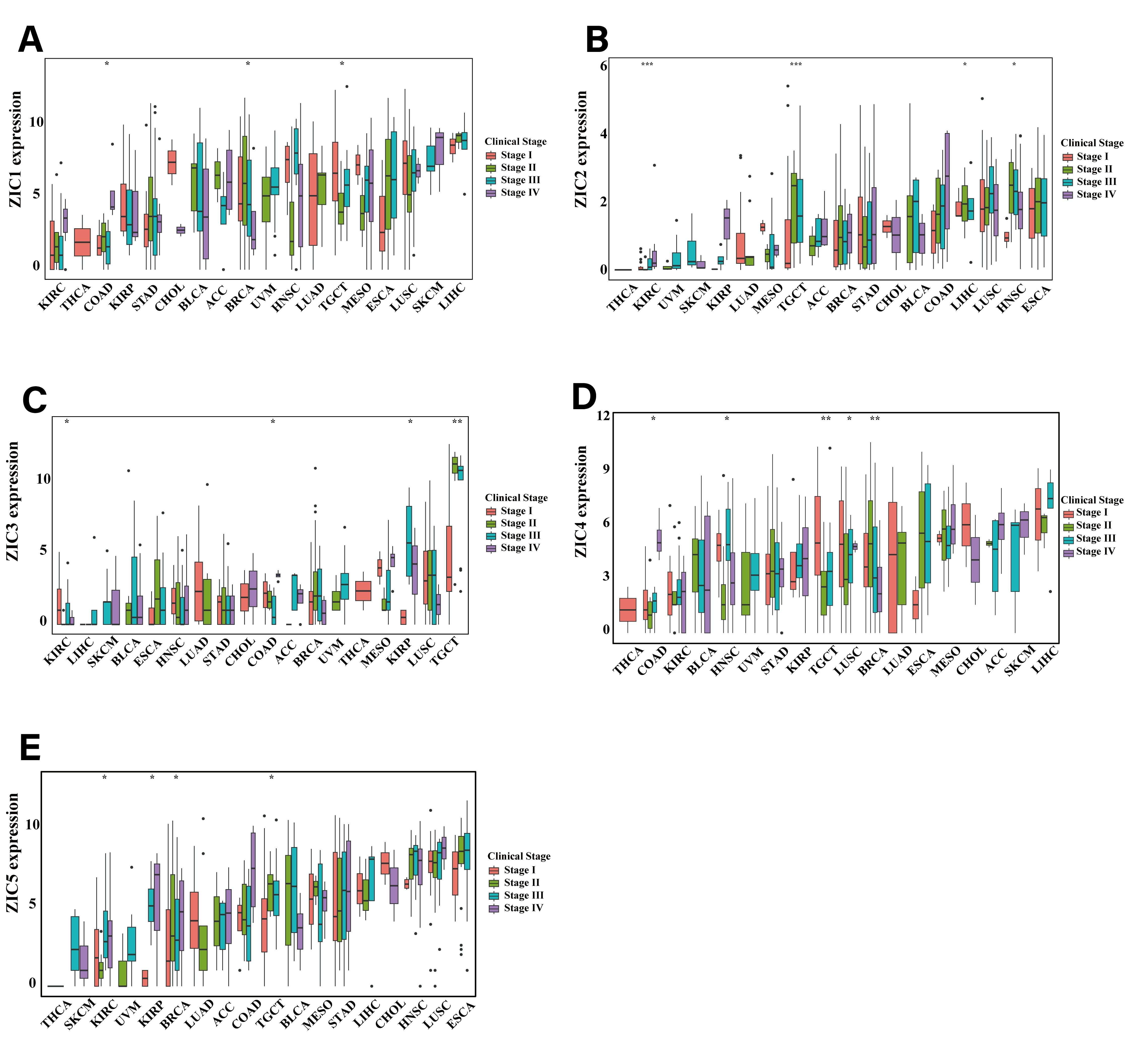

Supplement: Supplementary file 21 — Additional file 21. [file 12672_2026_5100_MOESM21_ESM.tif]
