# Supplementary material for: Multi-omics integration reveals pan-cancer roles of ZIC family genes in prognosis, immune microenvironment remodeling, and therapeutic vulnerability
Source: Discov Oncol. 2026 Apr 28;17:890. doi: 10.1007/s12672-026-05100-2 (PMC13253929; doi:10.1007/s12672-026-05100-2)
Supplement: Supplementary file 4 — Additional file 4. [file 12672_2026_5100_MOESM4_ESM.docx]

Supplementary Figure S1. Association between individual *ZIC* family genes and 33 distinct primary cancer types (A-E). Credit: [All images created by the authors].

Supplementary Figure S2. The *ZIC* expression levels in cancerous tissues were compared to those in their corresponding normal tissues (A-E). Exact *p*-values for all correlations are provided in Supplementary Table S4. *, *p* < 0.05; **, *p* < 0.01; ***, *p* < 0.001. Credit: [All images created by the authors].

Supplementary Figure S3. The mutation frequencies of specific *ZIC* genes were examined across (A-K) in a pan-cancer analysis and distinct cancer subtypes. Credit: [All images created by the authors].

Supplementary Figure S4. The relationship between SNVs and *ZIC* mRNA expression levels (A-E). Credit: [All images created by the authors].

Supplementary Figure S5. The relationship between various *ZIC* expression levels and TMB (A-E). *, *p* < 0.05; **, *p* < 0.01; ***, *p* < 0.001. Credit: [All images created by the authors].

Supplementary Figure S6. Correlation analysis of diverse *ZIC* exp. with MSI (A-E). *, *p* < 0.05; **, *p* < 0.01; ***, *p* < 0.001. Credit: [All images created by the authors].

Supplementary Figure S7. The association between the expression of each *ZIC* gene and the stages of pan-cancer patients (A-E). Exact *p*-values for all correlations are provided in Supplementary Table S4. *, *p* < 0.05; **, *p* < 0.01; ***, *p* < 0.001. Credit: [All images created by the authors].

Supplementary Figure S8. The association between *ZIC* exp. and patients response following initial treatment across pan-cancer (A-E). Exact *p*-values for all correlations are provided in Supplementary Table S4. CR, Complete response; PR, Partial response; PD, Progressive disease; SD, Stable disease *, *p* < 0.05; **, *p* < 0.01. Credit: [All images created by the authors].

Supplementary Figure S9. The correlation of ICG mRNA level with individual *ZIC* exp. levels across various tumors (A-E). Heatmaps illustrating the Spearman’s rank correlation between the mRNA expression levels of (A) *ZIC1*, (B) *ZIC2*, (C) *ZIC3*, (D) *ZIC4*, and (E) *ZIC5* and a panel of established ICGs across various cancer types. The color gradient indicates the correlation coefficient, where red denotes a positive correlation and blue represents a negative correlation. *, *p* < 0.05; **, *p* < 0.01; ***, *p* < 0.001. Credit: [All images created by the authors].

Supplementary Figure S10. (A) Single-cell data underwent quality control and visualization processes. (B) Correlation analysis was applied to RNA counts, mitochondrial content, and erythrocyte counts. (C) The clustering results were visualized using UMAP and tSNE. Credit: [All images created by the authors].
