# Supplementary material for: Multi-omics integration reveals pan-cancer roles of ZIC family genes in prognosis, immune microenvironment remodeling, and therapeutic vulnerability
Source: Discov Oncol. 2026 Apr 28;17:890. doi: 10.1007/s12672-026-05100-2 (PMC13253929; doi:10.1007/s12672-026-05100-2)
Supplement: Supplementary file 5 — Additional file 5. [file 12672_2026_5100_MOESM5_ESM.docx]

**Supplementary Methods**

**Western blotting**

Total protein from LIHC tissues and cell lines (LO2, HepG2, HuH-7, and Hep3B) was extracted using RIPA lysis buffer supplemented with protease inhibitors. Protein concentrations were quantified using a BCA Protein Assay Kit. Equal amounts of protein (20 μg per lane) were separated by 10% SDS-PAGE and transferred onto PVDF membranes. After blocking with 5% non-fat milk for 2 h at room temperature, the membranes were incubated with primary antibodies against ZIC2 (abcam, 1:1000) and GAPDH (abcam, 1:5000) at 4°C overnight. Following incubation with HRP-conjugated secondary antibodies, the protein bands were visualized using an enhanced chemiluminescence (ECL) detection system.

**Cell Counting Kit-8 (CCK-8) assay**

Cells (1 × 10^3^ per well) were seeded into 96-well plates, with three replicates per cell line. After attachment, 100 μL of a DMEM:CCK-8 (APEXBIO, K1018) mixture (10:1) was added per well, followed by a 2-h incubation and absorbance measurement at 450 nm. For pharmacological sensitivity assays, HCC cell lines (HepG2, HuH-7, and Hep3B) and the normal liver cell line LO2 were seeded into 96-well plates. After 24 h of adherence, the cells were treated with alisertib (MedChemExpress, USA) at a range of concentrations (0, 0.01, 0.05, 0.1, 0.5, 1, 5, and 10 μM) for 48 h. Subsequently, CCK-8 reagent was added to each well and incubated for 2 h at 37°C. The absorbance at 450 nm was measured using a microplate reader.
