# Supplementary material for: Multi-omics integration reveals pan-cancer roles of ZIC family genes in prognosis, immune microenvironment remodeling, and therapeutic vulnerability
Source: Discov Oncol. 2026 Apr 28;17:890. doi: 10.1007/s12672-026-05100-2 (PMC13253929; doi:10.1007/s12672-026-05100-2)
Supplement: Supplementary file 11 — Additional file 11. [file 12672_2026_5100_MOESM11_ESM.pdf]

ZYY-IRB-SOP-016(F)-002-03

伦理审查批件

批件号	IRB2025-YX-226-01		
项目名称	ZIC2的肝癌综合分析：利用多指标数据分析预后、免疫渗透和治疗意义		
项目来源	国家自然科学基金		
研究单位	天津医科大学基础医学院、天津医科大学总医院神经病学研究所		
主要研究者	于林、石翠娟		
审查类别	初始审查	审查方式	会议审查
审查日期	2025年05月29日	审查地点	天津医科大学总医院<br>第三住院楼4楼第二会议室
审查委员	王增光、戴向晨、张建宁、章志翔、刘强、杜亭亭、康春生、唐健、沈悦好、朱晓冬、李嵘、高明明		
批准文件	临床研究方案 （版本号：1.0 版本日期：2022.1.12）<br>免知情同意说明		

审查意见：

根据《涉及人的生命科学和医学研究伦理审查办法》（国科卫教发[2023]4号）、NMPA《药物临床试验质量管理规范(2020)》、《医疗器械临床试验质量管理规范(2022)》、WMA《赫尔辛基宣言》和CIOMS《人体生物医学研究国际道德指南》的伦理原则，经本伦理委员会审查，同意按所批准的临床研究方案、知情同意书、招募材料（如有）、受试者相关材料（如有）开展本项研究。

请遵循GCP原则、遵循伦理委员会批准的方案开展临床研究，保护受试者的健康与权利。

研究开始前，请申请人完成临床试验注册。

研究过程中若变更主要研究者，对临床研究方案、知情同意书、招募材料等的任何修改，请申请人提交修正案审查申请。

发生严重不良事件，请申请人及时提交严重不良事件报告。请按照伦理委员会规定的年度/定期跟踪审查频率，申请人在截止日期前1个月提交研究进展报告；申办者应当向组长单位伦理委员会提交各中心研究进展的汇总报告；当出现任何可能显著影响试验进行或增加受试者危险的情况时，请申请人及时向伦理委员会提交书面报告。

研究纳入了不符合纳入标准或符合排除标准的受试者，符合中止试验规定而未让受试者退出研究，给予错误治疗或剂量，给予方案禁止的合并用药等没有遵从方案开展研究的情况；或可能对受试者的权益、建康以及研究的科学性造成不良影响等违背GCP原则的情况，请申办者/监查员/研究者提交违背方案报告。

申请人暂停或提前终止临床研究，请及时提交暂停/终止研究报告。

完成临床研究，请申请人提交结题报告。

联系人与联系电话	常虹　　022-60363203
主任委员签字	[signature]
伦理委员会	天津医科大学总医院医学伦理委员会　（盖章）
日期	2025 年 5 月 29 日
